# Overexpression of the signaling coordinator *GAB2* can play an important role in acute myeloid leukemia progression

**DOI:** 10.1172/JCI195929

**Published:** 2025-08-07

**Authors:** Michael H. Kramer, Stephanie N. Richardson, Yang Li, Tiankai Yin, Nichole M. Helton, Daniel R. George, Michelle Cai, Sai Mukund Ramakrishnan, Casey D.S. Katerndahl, Christopher A. Miller, Timothy J. Ley

**Affiliations:** Washington University School of Medicine, Department of Medicine, Division of Oncology, St. Louis, Missouri, USA.

**Keywords:** Genetics, Oncology, Leukemias

## Abstract

Mutations that initiate acute myeloid leukemia (AML) can cause clonal expansion without transformation (clonal hematopoiesis). Cooperating mutations, usually in signaling genes, are needed to cause overt disease, but these may require a specific fitness state to be tolerated. Here, we show that nearly all AMLs arising in a mouse model expressing 2 common AML-initiating mutations (*Dnmt3a*^R878H^ and *Npm1*^cA^) acquired a single copy amplification of chromosome 7 (chr7), followed by activating mutations in signaling genes. We show that overexpression of a single gene on chr7 (*Gab2*, which coordinates signaling pathways) was tolerated in the presence of the *Npm1*^cA^ mutation, could accelerate the development of AML, and was important for the survival of fully transformed AML cells. *GAB2* is likewise overexpressed in many human AMLs with mutations in *NPM1* and/or signaling genes, and also in acute promyelocytic leukemia initiated by *PML::RARA*; the PML::RARA fusion protein may activate *GAB2* by directly binding to its 5′ flanking region. A similar pattern of GAB2 overexpression preceding mutations in signaling genes has been described in other human malignancies. *GAB2* overexpression may represent an oncogene-driven adaptation that facilitates the action of signaling mutations, suggesting an important (and potentially targetable) missing link between the initiating and progression mutations associated with AML.

## Abstract 2

This study identified overexpression of the signaling-coordinator *GAB2* as a progression event that may mechanistically link initiating and cooperating mutations in AML and other cancers.

## Graphical abstract

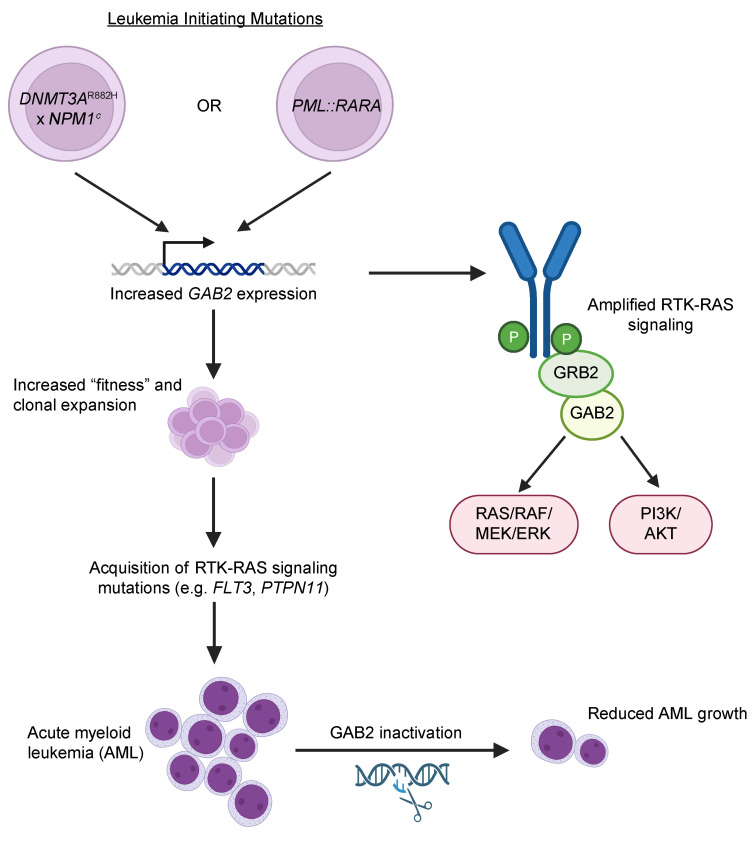

## Introduction

Acute myeloid leukemia (AML) is the most common acute leukemia in adults (1), affecting approximately 20,800 people in the United States in 2024. The prognosis for many patients remains poor, with 5-year overall survival rates of approximately 30% (2). AML is initiated by mutations that are subsequently found in all of the subclones of a given tumor (3). In some patients, these initiating mutations can be detected several years prior to the development of overt disease (“clonal hematopoiesis”); progression to overt disease is associated with the acquisition of additional cooperating mutations (4, 5). These cooperating mutations often cause activated proliferative signaling (e.g., caused by altered activity of *FLT3*, *PTPN11*, *KRAS*, *NRAS*, *KIT*, *NF1*, or *CBL*) (6–8). However, the links and dependencies between initiating mutations and progression mutations are not well understood.

*DNMT3A* and *NPM1* are 2 of the most frequently mutated genes in AML, each affecting 20%–30% of patients; these co-occur more frequently than expected by chance, with approximately 15% of patients with AML having mutations in both genes (7–9). When present, mutations in *DNMT3A* and/or *NPM1* nearly always co-occur in the founding clone (10).

*DNMT3A* is 1 of 2 major de novo DNA methyltransferases responsible for methylation of cytosine in CpG dinucleotides (11). The most common *DNMT3A* mutation in human AML is a heterozygous R882H missense substitution (R878H in mice); this dominant negative mutation leads to an approximately 80% reduction in DNMT3A activity, causing focal, canonical DNA hypomethylation at thousands of regions across the genome (9, 12–14).

Nucleophosmin (NPM1) is a nucleolar scaffolding protein (15). Falini and colleagues first described heterozygous frameshift mutations near the 3′ end of this gene (*NPM1*^c^), resulting in a new nuclear export signal and cytoplasmic mislocalization (16–18). *NPM1*^cA^ (a duplication of TCTG) is the most common subtype, occurring in 75%–80% of cases (18–20). Relocalization of mutant NPM1 to the nucleolus induces the differentiation of AML cells, suggesting that mislocalization is important for leukemogenesis (19, 21). While recent studies suggest that *NPM1*^c^ may be part of a nuclear transcription factor complex that activates HOX genes and MEIS1 (22, 23), many of the details of how *NPM1*^c^ initiates AML remain unclear (21).

Patients with AML with *NPM1* and *DNMT3A* mutations usually acquire cooperating mutations in signaling genes. In one large study (8), 85% (193 of 228) of AML samples with both *NPM1* and *DNMT3A* mutations contained an additional, cooperating mutation in an RTK/RAS pathway gene (RTK/RAS signaling), compared with 52% (505 of 975) of AML samples with neither mutation (*P* < 10^–21^, by χ^2^). Patients with mutant *NPM1* and WT *DNMT3A* had a slightly lower (but still increased) likelihood of a mutation in an RTK/RAS signaling gene (76% of cases; 158 of 208; *P* < 10^–17^); patients with mutant *DNMT3A* and WT *NPM1* had RTK/RAS signaling mutations at frequencies similar to those seen in AMLs without either *DNMT3A* or *NPM1* mutations (48%; 62 of 129), suggesting that *NPM1* is the main driver of the association with RTK/RAS signaling mutations. The mechanistic links between initiating mutations in *NPM1* and RTK/RAS signaling mutations are not well understood.

Previous studies performed by Trowbridge and colleagues established a conditional mouse model for hematopoietic cell–specific induction of the *Npm1*^cA^ mutation, which, in combination with the *Dnmt3a*^R878H^ mutation, induces progression to AML with additional spontaneous mutations in RTK/RAS signaling genes that are strikingly similar to those found in human AMLs (24). Follow-up studies of this model identified hematopoietic stem cells (HSCs) as the cell of origin and defined changes in gene expression and chromatin accessibility (25).

In this study, a similar model served as a clinically relevant, experimentally tractable foundation to study the molecular link between founding and cooperating mutations in AML. We identified overexpression of *Gab2*, the gene encoding the signaling nexus protein GAB2 (via amplification of murine chromosome 7 [chr7]) as a nearly ubiquitous progression event that depends on preexisting, initiating mutations in *Npm1* and *Dnmt3a* and occurs prior to acquisition of RTK/RAS signaling mutations. Coupled with functional studies and data from human tumors, these observations nominate GAB2 as a potentially targetable missing link between AML initiating mutations and downstream RTK/RAS signaling mutations associated with AML progression.

## Results

### Mice with Dnmt3a^R878H^ and Npm1^cA^ mutations spontaneously develop AML with amplification of chr7 and cooperating signaling mutations.

To investigate the molecular mechanisms associated with AML progression in the presence of *Dnmt3a*^R878H^ and *Npm1*^cA^ initiating mutations, we utilized a mouse model with a heterozygous germline *Dnmt3a*^R878H/+^ mutation (26) and a conditional *Npm1*^cA/+^ allele (24, 27), along with a tamoxifen-inducible flippase transgene, used to activate the *Npm1* mutation. Bone marrow from these mice was transplanted into recipient mice, followed by administration of tamoxifen after engraftment to create hematopoietic-specific *Dnmt3a*^R878H/+^ × *Npm1*^cA/+^ mice. Expression of both mutant alleles in virtually all bone marrow cells was confirmed by RNA-Seq (data not shown). Consistent with previous reports, these mice spontaneously developed myelomonocytic AML within 6–15 months; all 9 tested AMLs rapidly caused fatal disease when transplanted into secondary recipients (data not shown).

**Figure 1:**
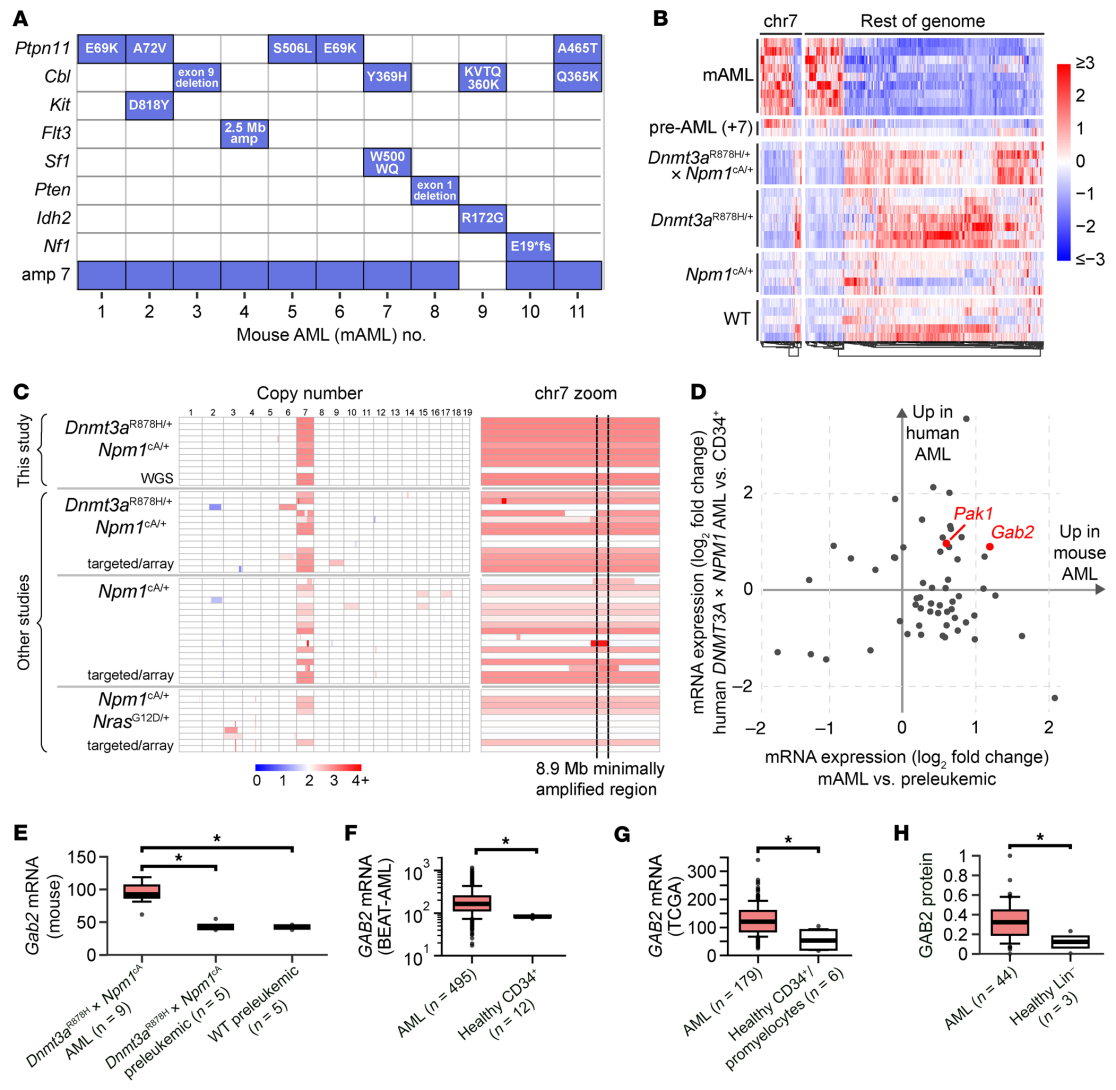
Mice with *Dnmt3a*^R878H^ and *Npm1*^cA^ mutations spontaneously develop AML with amplification of chr7 and cooperating signaling mutations. (**A**) Whole-genome sequencing of 11 AMLs arising in *Dnmt3a*^R878H/+^ *× Npm1*^cA/+^ mice (mAMLs) revealed mutations in cancer driver genes and amplification of chr7 in 10 of 11 tumors. amp, amplification. (**B**) RNA expression (*z* scores) is shown for DEGs from mAML versus preleukemic *Dnmt3a*^R878H/+^ *× Npm1*^cA/+^ samples (FDR < 0.01). Shown are mAML samples (*n* = 9), pre-AML murine bone marrow samples (nontransplantable clonal expansions with +7 in *Dnmt3a*^R878H/+^ *× Npm1*^cA/+^ mice; *n* = 2), and preleukemic murine bone marrow samples of the indicated genotypes. Genes are split by genomic location (chr7 vs. the rest of the genome) and hierarchically clustered. (**C**) Left: Copy number changes in mAMLs (this study), panel sequencing data from a similar mouse model (24), and aCGH of an *Npm1^cA^*-driven tumor model (28). Right: The minimally amplified region identified on chr7. WGS, whole-genome sequencing. (**D**) mRNA expression of genes on the chr7 minimally amplified region and their human orthologs. The *x* axis represents mouse mAML tumors compared with *Dnmt3a*^R878H/+^ *× Npm1*^cA/+^ preleukemic samples from this study; the *y* axis represents human AML tumors with *DNMT3A*^R882C/H^ and *NPM1*^c^ mutations compared with healthy CD34^+^ cells (75). (**E**–**G**) mRNA expression of *Gab2* in mAML versus controls in this mouse model (**E**) and in patients from the BEAT-AML study (**F**) (*y* axis: log_10_ scale to show the range in the presence of high *Gab2* AML samples) (75) or TCGA (**G**) (7). (**H**) Protein abundance in AMLs versus healthy lineage-depleted bone marrow samples, measured by tandem-mass-tag mass spectrometry, scaled to between 0 and 1 for display (43). *P < 0.05, by 2-tailed *t* test. Boxes show median (line) and extend from the 25th to 75th inter-quartile range (IQR), with whiskers showing 1.5X the IQR, and outlier points as shown outside the whiskers (**E**–**H**), adjusted for multiple hypothesis testing in **E** using the Benjamini-Hochberg method.

We characterized preleukemic bone marrow samples and 11 independent spontaneous AMLs that developed in these doubly mutant mice (mAMLs 1–11). Whole-genome sequencing revealed that each tumor acquired 1 or more human AML-like cooperating mutations, including RTK/RAS signaling mutations in *Ptpn11* (E69K, A72V, A465T, or S506L), *Kit* (D818Y), *Cbl* (Y369H, Q365K, or exon 9 deletion), *Nf1* (frameshift), and a focal (2.5 Mbp) amplification of chromosome 5 that included *Flt3*, associated with more than 20-fold overexpression of that gene (Figure 1A and Supplemental Table 1; supplemental material available online with this article; https://doi.org/10.1172/JCI195929DS1). No gene fusion events were detected. Remarkably, 10 of 11 AMLs contained an amplification of murine chr7, usually as the only structural variant. Furthermore, we identified 2 nonleukemic mice for which whole-genome sequencing revealed amplification of chr7 (+7) and no other cooperating mutations in identifiable driver genes. One of these clonal expansions was tested twice in secondary transplantation assays and did not lead to fatal disease when transplanted into recipients, suggesting that +7 is an intermediate step in AML progression. Characterization of preleukemic bulk mRNA-Seq data demonstrated little change in the preleukemic state for several months, followed by dramatic and canonical shifts in transcription after transformation (Figure 1B). A total of 821 genes were differentially expressed (FDR < 0.01) in the AML samples compared with preleukemic *Dnmt3a*^R878H/+^ *× Npm1*^cA/+^ samples; 211 genes had increased expression in AML samples, and 610 genes showed decreased expression. Gene Ontology (GO) enrichment analysis showed that gene sets with increased expression in the AML samples include “regulation of programmed cell death” (FDR < 0.001), “tumor necrosis factor superfamily cytokine production” (FDR < 0.01), “interleukin 6 production” (FDR = 0.01), and “kinase activity” (FDR < 0.001), while gene sets with decreased expression in the AML samples include “myeloid cell differentiation” (FDR < 1 × 10^–32^) and “lymphocyte activation” (FDR < 1 × 10^–22^) (Supplemental Table 2).

We next analyzed panel sequencing data from a published study using a similar doubly mutant mouse model from Trowbridge and colleagues (24), and array comparative genomic hybridization (aCGH) data from a different *Npm1*^cA^-driven tumor model described by Vassiliou and colleagues (28); these AMLs also developed complete or partial amplification of chr7 in 7/8 and 10/15 samples, respectively. Parts of mouse chr7 are syntenic with regions from human chromosomes 1, 2, 6, 10, 11, 12, 15, 16, 19, and X, so identifying a single human structural event that recapitulates +7 in the mouse is not possible. However, through integration of data from all tumor models, we identified a minimally amplified 8.9 Mbp region on chr7 (Figure 1C) containing 206 protein-coding genes (including 97 olfactory receptor genes; Supplemental Table 3). The minimally amplified region is syntenic to 6 different regions of human chr11 (in 6 separate blocks, ranging from 11p15.4 to 11q14.1). Amplification of chr7 was linked to increased expression of many genes: 46.0% (97 of 211) of the genes with significantly increased expression were found on chr7, whereas only 3.8% (23 of 610) of the genes with significantly decreased expression were on chr7.

### Identification of Gab2 as a relevant gene in the minimally amplified region on chr7.

The minimally amplified region contains 206 genes, 97 of which encode olfactory receptors that are unlikely to be relevant for AML pathogenesis. While functionalizing all potential contributors to positive selection would be challenging, we prioritized genes for functional characterization using the following criteria: (a) evidence of similar overexpression in human AML samples with *DNMT3A*^R882^ and *NPM1*^c^ mutations (Figure 1D); (b) evidence of overexpression in conjunction with the amplification; (c) evidence of cancer relevance in the OncoKB list of putative cancer-related genes (29); and (d) evidence of dependency in myeloid cells: CRISPR perturbations of these genes in cell lines reported by DepMap showed some evidence of dependency in myeloid cell lines (30, 31). Three genes met at least 3 of these criteria: *GAB2*, *PAK1*, and *INPPL1*; *GAB2* was the only gene that met all 4.

Additional lines of evidence suggested that Grb2-associated binding protein 2 (*GAB2*) should be a leading candidate, due in part to the elevated expression of *GAB2* in AML samples observed at both the mRNA and protein levels in multiple datasets (Figure 1, E–H). *PAK1* was also selected for further study, given its presence in the minimally amplified region, increased expression in both human and mouse AML (Figure 1D), and recent work suggesting a role in AML pathogenesis (32, 33). *INPPL1* did not have the same strength of evidence and was therefore not studied further.

**Figure 2:**
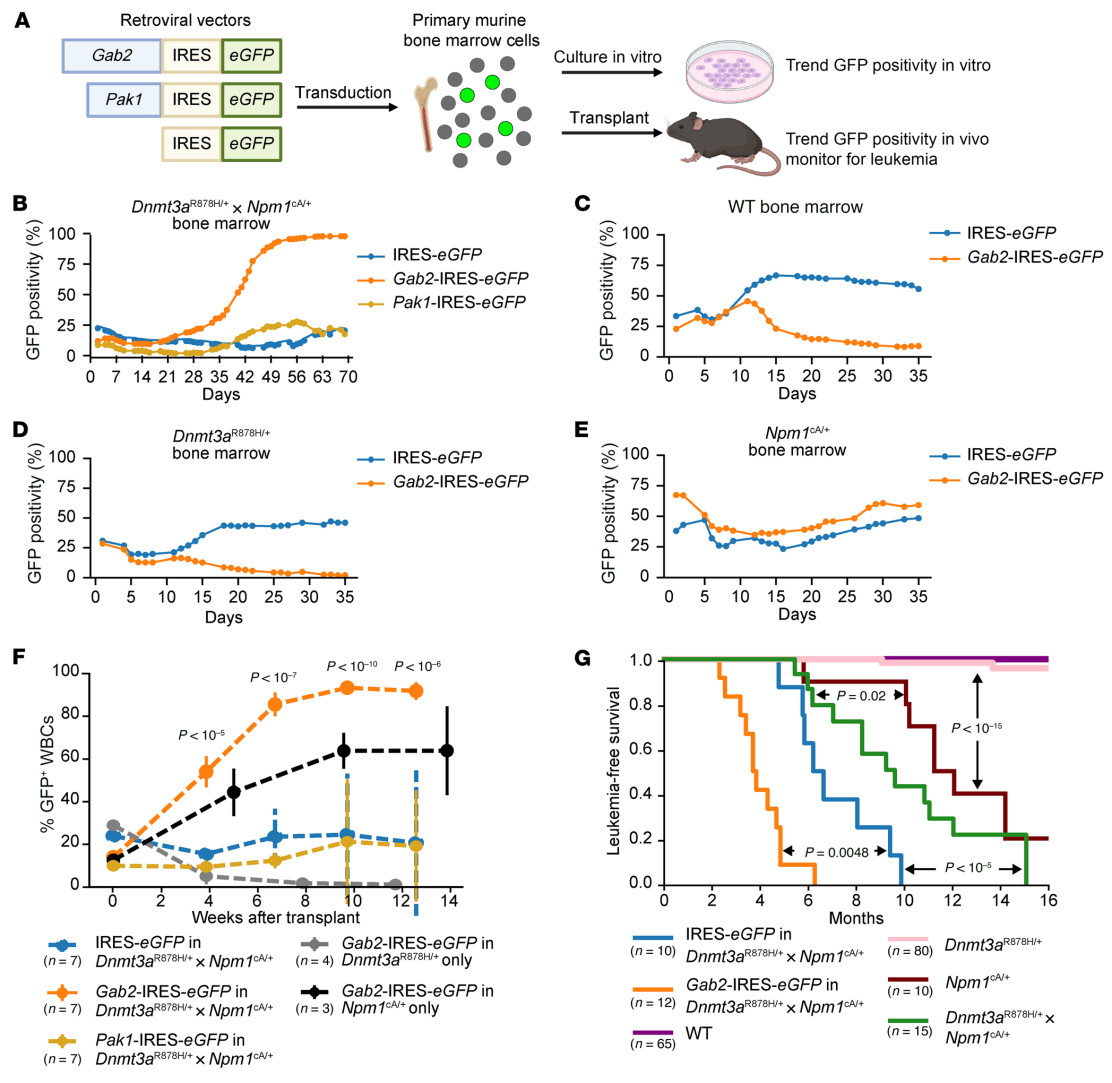
Retroviral overexpression of *Gab2* causes the expansion of HSPCs and accelerates the development of AML in the presence of *Npm1*^cA^ and *Dnmt3a*^R878H^ mutations. (**A**) Retroviral vectors were transduced into lineage-depleted primary murine CD45.2^+^ bone marrow cells to create a mixed population of transduced (GFP^+^) and untransduced (GFP^–^) cells. Cells were split into 2 groups, with 1 maintained in vitro and 1 transplanted into CD45.1^+^ recipient mice. The fraction of GFP^+^ cells was measured over time using flow cytometry. This illustration was created in BioRender. (**B**) Overexpression of *Gab2* in *Dnmt3a*^R878H/+^ *× Npm1*^cA/+^ marrow shows a growth advantage in vitro, with GFP^+^ (*Gab2-*overexpressing) cells expanding over time. No effect was observed in cells transduced with the IRES-*eGFP* or *Pak1*-IRES-*eGFP* vectors. Representative data from 1 of 4 biological replicates are shown. (**C** and **D**) Overexpression of *Gab2* in (**C**) WT or (**D**) *Dnmt3a*^R878H/+^ bone marrow caused a selective disadvantage compared with IRES-*eGFP* vector–only cells or untransduced cells. Data shown in **C** and **D** are representative of 1 of 2 biological replicates. (**E**) Overexpression of *Gab2* in *Npm1*^cA/+^ bone marrow did not show a marked advantage or disadvantage compared with IRES-*eGFP* vector or untransduced cells. Data shown are representative of 1 of 2 biological replicates. (**F**) GFP positivity of CD45.2^+^ peripheral WBCs was measured by flow cytometry in CD45.1 mice transplanted with retrovirally transduced cells of the indicated genotypes. Dots show mean values at each time point, and error bars show SD. *P* values were calculated at each time point for the *Gab2*-IRES-*eGFP* curve using a 2-sided, 1-sample *t* test for change from baseline compared with a null hypothesis of a mean change of 0, with Benjamini-Hochberg multiple hypothesis correction. (**G**) Mice of the indicated genotypes were monitored for AML development. Retroviral overexpression of *Gab2* in the *Dnmt3a*^R878H/+^ *× Npm1*^cA/+^ background significantly accelerated AML development. *P* values were determined by pairwise log-rank test.

To determine whether increased expression of *Gab2* and/or *Pak1* was relevant for AML progression in this model, we prepared retroviral constructs expressing candidate cDNAs (with an IRES-*eGFP* tag) and transduced them into lineage-depleted, nonleukemic bone marrow cells from WT, *Dnmt3a*^R878H/+^ only*, Npm1*^cA/+^ only*,* or *Dnmt3a*^R878H/+^ *× Npm1*^cA/+^ doubly-mutant mice, creating an initial mixture of transduced (GFP^+^) and nontransduced (GFP^–^) cells (Figure 2A). GAB2 and PAK1 protein overexpression was confirmed by Western blotting (Supplemental Figure 1). These mixtures of cells were maintained in vitro, and GFP positivity was measured over time by flow cytometry. In *Dnmt3a*^R878H/+^ × *Npm1*^cA/+^ bone marrow, we observed marked expansion of *Gab2*-overexpressing, but not *Pak1*-overexpressing, cells over time, with GFP positivity increasing in culture approximately 2 weeks after retroviral transduction and eventually reaching greater than 95% GFP positivity (Figure 2B). In contrast, *Gab2* overexpression showed a selective disadvantage in WT bone marrow (Figure 2C) and in bone marrow cells with the *Dnmt3a*^R878H/+^ mutation only (Figure 2D). In bone marrow cells with the *Npm1*^cA/+^ mutation, *Gab2* overexpression did not affect cell growth in vitro (Figure 2E).

The transduced cell populations were also transplanted into recipient mice to monitor for effects of *Gab2* or *Pak1* overexpression in vivo. Consistent with the in vitro results, overexpression of *Gab2* (but not *Pak1*) in vivo induced significant expansion of hematopoietic cells from *Dnmt3a*^R878H/+^ *× Npm1*^cA/+^ mice (and, to a lesser extent, from *Npm1*^cA/+^ mice), but not *Dnmt3a*^R878H/+^ mice, suggesting that the mutations in *Npm1* and *Dnmt3a* synergize to account for this phenotype (Figure 2F). Furthermore, overexpression of *Gab2* in *Dnmt3a*^R878H/+^ *× Npm1*^cA/+^ bone marrow led to the development of AML in 12 of 12 engrafted mice, with a median latency of 3.7 months (range, 2.3–6.3); 10 mice engrafted with an IRES-*eGFP*–only vector developed AML with a longer median latency of 6.2 months (range, 4.8–9.9, *P* = 0.0048; Figure 2G). The IRES-*eGFP* vector alone probably facilitated AML development due to its well-described ability to cause insertional mutations, which can be selected for if they activate oncogenes or disrupt tumor suppressors (34–37). Overexpression of *Gab2* in *Npm1*^cA/+^ bone marrow cells led to the development of AML in 7 of 7 engrafted mice with a median latency of 4.5 months, compared with a latency of 7.5 months with the IRES-*eGFP*–only vector (*P* = 0.12; Supplemental Figure 2A). Taken together, these data suggest that the *Npm1*^cA^ and *Dnmt3a*^R878H^ mutations synergized to create a permissive environment that selected for *Gab2* overexpression as an important step in leukemic transformation.

Whole-genome sequencing of 9 AMLs that developed with the *Gab2*-IRES-*eGFP* retrovirus in *Dnmt3a*^R878H/+^ *× Npm1*^cA/+^ bone marrow (GAB2 mAMLs 1–9) revealed amplification of the entirety of chr7 in 5 of 9 cases, suggesting that additional genes on chr7 may still be relevant for some AMLs arising in this model. Three tumors had no amplification of chr7, and 1 tumor had partial amplification of chr7 (Supplemental Figure 2B). Strikingly, this partial amplification did not include the region containing *Gab2*, suggesting that *Gab2* retroviral overexpression diminished the selective pressure for *Gab2* amplification. Additional cooperating mutations in known cancer genes (including *Nf1*, *Ep300*, *Notch1*, *Sbds*, and *Ets1*) were identified in 5 of the 9 GAB2 mAMLs (Supplemental Table 4). Only 1 of 9 GAB2 mAMLs contained a cooperating mutation in an RTK/RAS signaling gene (*Nf1* frameshift), suggesting that the level of *Gab2* overexpression in these AMLs (12.4-fold higher than mAMLs developing spontaneously) may reduce the need for further RTK/RAS signaling mutations. There are 169 protein-coding genes (including 97 olfactory receptor genes; Supplemental Table 5) on the remaining 4.6 Mbp minimally amplified region. Although 1 or more additional genes in this region could also be relevant for AML progression, many additional studies would be required to identify it. For this study, we therefore decided to limit our focus to the study of *Gab2* and its clearcut ability to accelerate AML development.

### Increased Gab2 expression does not cause major transcriptional or immunophenotypic changes.

**Figure 3:**
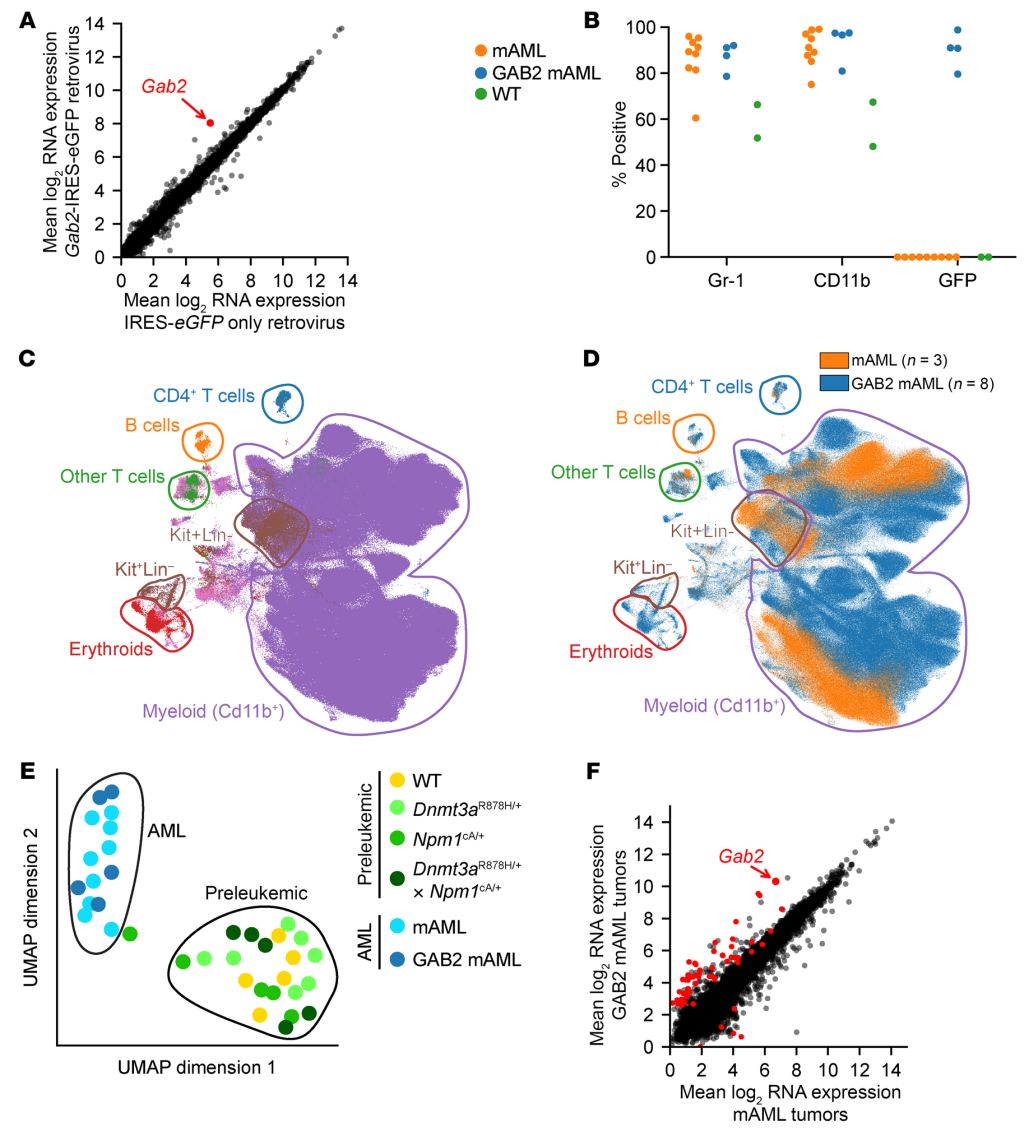
Increased *Gab2* expression does not cause major transcriptional or immunophenotypic changes in AML. (**A**) Preleukemic *Dnmt3a*^R878H/+^ *× Npm1*^cA/+^, lineage-depleted murine bone marrow cells were transduced with retroviruses expressing *Gab2*-IRES-*eGFP* or IRES-*eGFP* only. Cells were maintained in vitro and harvested 1 or 4 weeks after transduction. GFP^+^ cells were then enriched by flow cytometry, and bulk RNA-Seq was performed. Two biological replicates were performed for each condition and time point (*n* = 4 per group). Each dot represents 1 gene. *Gab2* (red) is the only DEG (FDR < 0.05). (**B**) Flow cytometry was performed on bone marrow samples from leukemic mice and WT bone marrow samples. The percentage of CD45.2^+^ cells positive for each of the markers indicated on the *x* axis is shown. mAML denotes murine AMLs developing spontaneously in *Dnmt3a*^R878H/+^ *× Npm1*^cA/+^ mice; GAB2 mAML denotes murine AMLs developing with *Gab2* retroviral overexpression in *Dnmt3a*^R878H/+^ *× Npm1*^cA/+^ mice. (**C** and **D**) UMAP of cells from leukemic mice with mAML or GAB2 mAML, characterized using a 23-color hematopoietic flow cytometry panel. Note that the majority of cells in all samples are Cd11b^+^ myeloid cells. Cells are colored by cell type in **C** and by sample type in **D**. (**E**) UMAP created from bulk mRNA-Seq data collected from murine bone marrow samples of the indicated types (annotated by color). Preleukemic and leukemic cells formed mostly distinct clusters, whereas mAMLs and GAB2 mAMLs colocalized. (**F**) Mean expression levels of all genes as determined by bulk mRNA-Seq of GAB2 mAML tumors (*n* = 5) and mAML tumors (*n* = 9). DEGs (FDR < 0.01) are colored red. See Supplemental Table 6 for the list of 57 DEGs.

To evaluate the downstream effects of *Gab2* overexpression, we performed bulk mRNA-Seq of preleukemic primary murine *Dnmt3a*^R878H/+^ *× Npm1*^cA/+^ hematopoietic stem and progenitor cells (HSPCs) with retrovirally overexpressed *Gab2* (using a *Gab2*-IRES-*eGFP* vector), compared with those transduced with a vector containing IRES-*eGFP* only. Murine bone marrow cells were harvested, transduced with each retrovirus, and then maintained in vitro and harvested at 1 and 4 weeks (before and after marked selection for *Gab2*-overexpressing cells was observed; see Figure 2B). At each time point, GFP^+^ cells were purified for RNA-Seq. When we compared *Gab2*-overexpressing cells with those transduced with GFP only, the only differentially expressed gene (DEG) (defined as having an FDR < 0.05) between the samples was *Gab2* itself, which had 5.9-fold higher expression on average in the samples with *Gab2* retrovirus (FDR = 0.017, Figure 3A). Evaluation of the week 4 samples (after *Gab2-*overexpressing cells had outgrown the untransduced cells) revealed no additional DEGs; *Gab2* mRNA itself had 4.1-fold higher expression on average in the samples with retrovirally overexpressed *Gab2* (normalized expression values of 257 and 139 counts per million with *Gab2*-IRES-*eGFP,* versus 47 and 49 counts per million with IRES-*eGFP* alone; *P* = 0.00026). These data suggest that the downstream effects of *Gab2* overexpression were primarily posttranscriptional in preleukemic cells.

We next characterized the fully transformed AML samples using multiparameter, spectral flow cytometry with a 23-marker hematopoietic panel (38). Immunophenotypically, we found that most cells in all AML samples were myelomonocytic, expressing Cd11b and Gr-1 on the majority of bone marrow cells (Figure 3B). Using the uniform manifold approximation and projection (UMAP) algorithm (39, 40) to visualize cells based on their immunophenotypes, we found that cells primarily aggregated by cell type (as defined by canonical surface markers) and AMLs with retrovirally overexpressed *Gab2* (GAB2 mAML) did not clearly separate from those that developed spontaneously (mAML; Figure 3, C and D).

We next characterized the retrovirally transduced GAB2 mAMLs using RNA-Seq. We used the UMAP procedure to visualize the global transcriptional signatures of the GAB2 mAMLs, compared with mAMLs, and preleukemic bone marrow of various genotypes (WT, *Dnmt3a*^R878H/+^, *Npm1*^cA/+^, and *Dnmt3a*^R878H/+^ *× Npm1*^cA/+^). Samples clustered primarily into preleukemic and leukemic groups, with the AMLs intermixed regardless of *Gab2* retroviral status (Figure 3E); hierarchical clustering of transcriptional profiles gave a similar result, with preleukemic samples forming a distinct group separate from both mAML and GAB2 mAML samples (Supplemental Figure 3). A direct comparison of AMLs with and without *Gab2* retroviral overexpression revealed only 57 DEGs (FDR < 0.01) between these samples, and we observed no enrichment for pathways known to be relevant for oncogenesis (Figure 3F and Supplemental Table 6). AMLs arising with retrovirally induced GAB2 overexpression are therefore highly similar (both transcriptionally and immunophenotypically) to those with GAB2 overexpression acquired via spontaneous chr7 amplification; AML latency is shortened with GAB2 overexpression, but the features of AML are highly conserved.

### The GAB2 protein interactome is shaped by Dnmt3a^R878H^ and Npm1^cA^ mutations.

**Figure 4:**
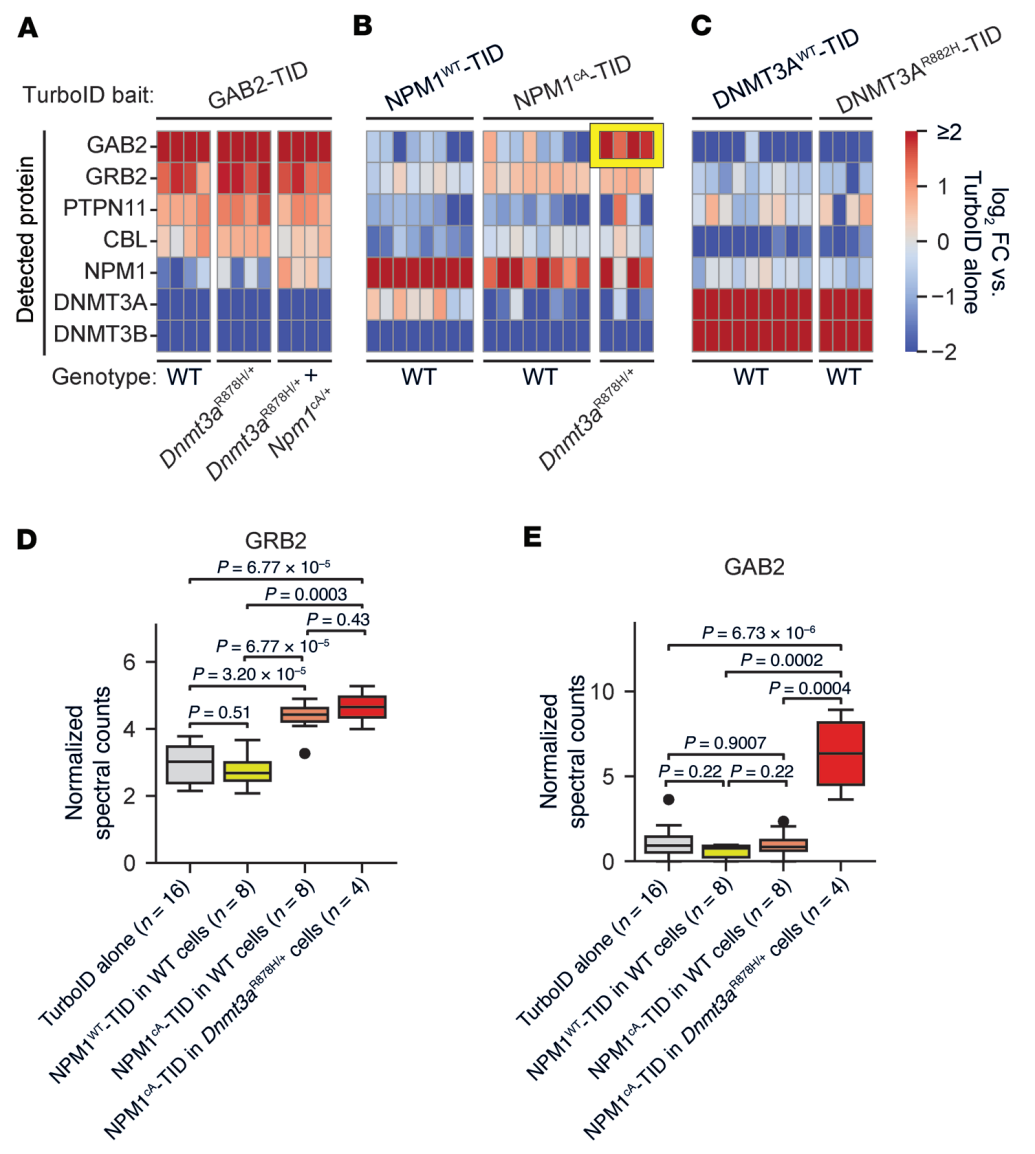
The GAB2 protein interactome is shaped by *Dnmt3a*^R878H^ and *Npm1*^cA^ mutations. (**A**–**C**) Protein proximity data derived from TurboID (TID) expression performed in primary, lineage-depleted murine bone marrow cells of the indicated genotypes. Each box represents a single replicate, colored according to the log_2_ fold change (FC) of spectral counts detected compared with the mean spectral counts detected in control samples using TurboID alone as the bait. As expected, each bait was self-labeling, and known interactions were detected, including for GAB2 (with GRB2, PTPN11, and CBL) and for DNMT3A (with DNMT3B). Yellow box highlights interactions with GAB2 and NPM1^cA^ observed in *Dnmt3a*^R878H/+^ cells. (**D** and **E**) Normalized GRB2 (**D**) and GAB2 (**E**) spectral counts detected in TurboID proximity labeling experiments. The *x* axis indicates the bait used and the genotype of the primary murine bone marrow cells transduced. Boxes show the median (line) and extend from the 25th to 75th IQR, with whiskers showing 1.5 times the IQR and outlier points as shown outside the whiskers. *P* values were calculated by 2-sided, 2-sample *t* test and adjusted for multiple hypothesis correction using the Benjamini-Hochberg method. Note that NPM1^cA^ (but not WT NPM1) shows an interaction with GRB2 in WT and *Dnmt3a*^R878H/+^ cells but interacts with GAB2 only in *Dnmt3a*^R878H/+^ cells.

To determine how the initiating mutations in this mouse model may create an environment that enables *Gab2* overexpression, we performed experiments using the TurboID system (41) to label intracellular proteins that are in close proximity to the GAB2, NPM1^WT^, NPM1^cA^, DNMT3A^WT^, or DNMT3A^R882H^ proteins (“baits”) in primary murine HSPCs. We used a *TurboID* cDNA fused in-frame to the N- or C-terminus of each bait in an MSCV-based retroviral vector and transduced these vectors into primary murine bone marrow HSPCs from relevant genotypes (WT, *Dnmt3a*^R878H/+^, or *Dnmt3a*^R878H/+^ × *Npm1*^cA+^). *TurboID* cDNA alone was used as a control for nonspecific biotin labeling of proteins. The TurboID biotin ligase is self-labeling, so any bait protein fused to it should be labeled and enriched by streptavidin bead pulldowns. As expected, we observed self-labeling of GAB2, NPM1, and DNMT3A in the appropriate samples (Figure 4, A–C). We also observed the expected interactions of GAB2 with some of its known partners, including GRB2, PTPN11, and CBL (Figure 4A). As another positive control, DNMT3A interacted with its known partner DNMT3B (Figure 4C). Importantly, we found that NPM1^cA^ (but not NPM1^WT^) had significant interaction with the GAB2-binding partner GRB2 in all genotypes (Figure 4, B and D), but with GAB2 itself only in the presence of the *Dnmt3a*^R878H^ mutation (Figure 4E).

We next evaluated whether GAB2 interactions with any of its downstream signaling partners were dependent on preexisting mutations in either *Dnmt3a* or *Npm1*. Indeed, we found that interactions between GAB2 and several PI3K proteins (PIK3CA, PIK3CB, PIK3CD) were stronger in the preleukemic *Dnmt3a*^R878H/+^ mutant cells compared with WT cells (Supplemental Figure 4A). We also noted stronger interactions between GAB2 and the regulatory subunit PIK3R1, and with PI4KA, in the preleukemic *Dnmt3a*^R878H/+^ × *Npm1*^cA/+^ mutant cells, compared with cells with 1 or neither mutation (Supplemental Figure 4B). None of the genes with genotype-dependent protein interactions with NPM1^cA^ or GAB2 in preleukemic HSPCs (including GAB2 itself) showed significant changes in mRNA expression across preleukemic genotypes (Supplemental Figure 5, A–F); only PIK3CB showed small but statistically significant increased protein expression (as measured by tandem-mass-tag mass spectrometry; see below) in *Dnmt3a*^R878H/+^ *× Npm1*^cA/+^ bone marrow compared with WT bone marrow (but no change in *Dnmt3a*^R878H/+^ bone marrow compared with WT cells; Supplemental Figure 5, G–K). Together, these data suggest that initiating mutations in *Dnmt3a* and *Npm1* may cooperate to shape the interactions of GAB2 with downstream signaling proteins; it is not yet clear whether this finding contributes to the selective advantage of *Gab2* overexpression in preleukemic cells with mutant *Npm1* and *Dnmt3a*.

### Some RTK/RAS signaling proteins that interact with NPM1^cA^ have increased protein abundance after leukemic transformation.

**Figure 5:**
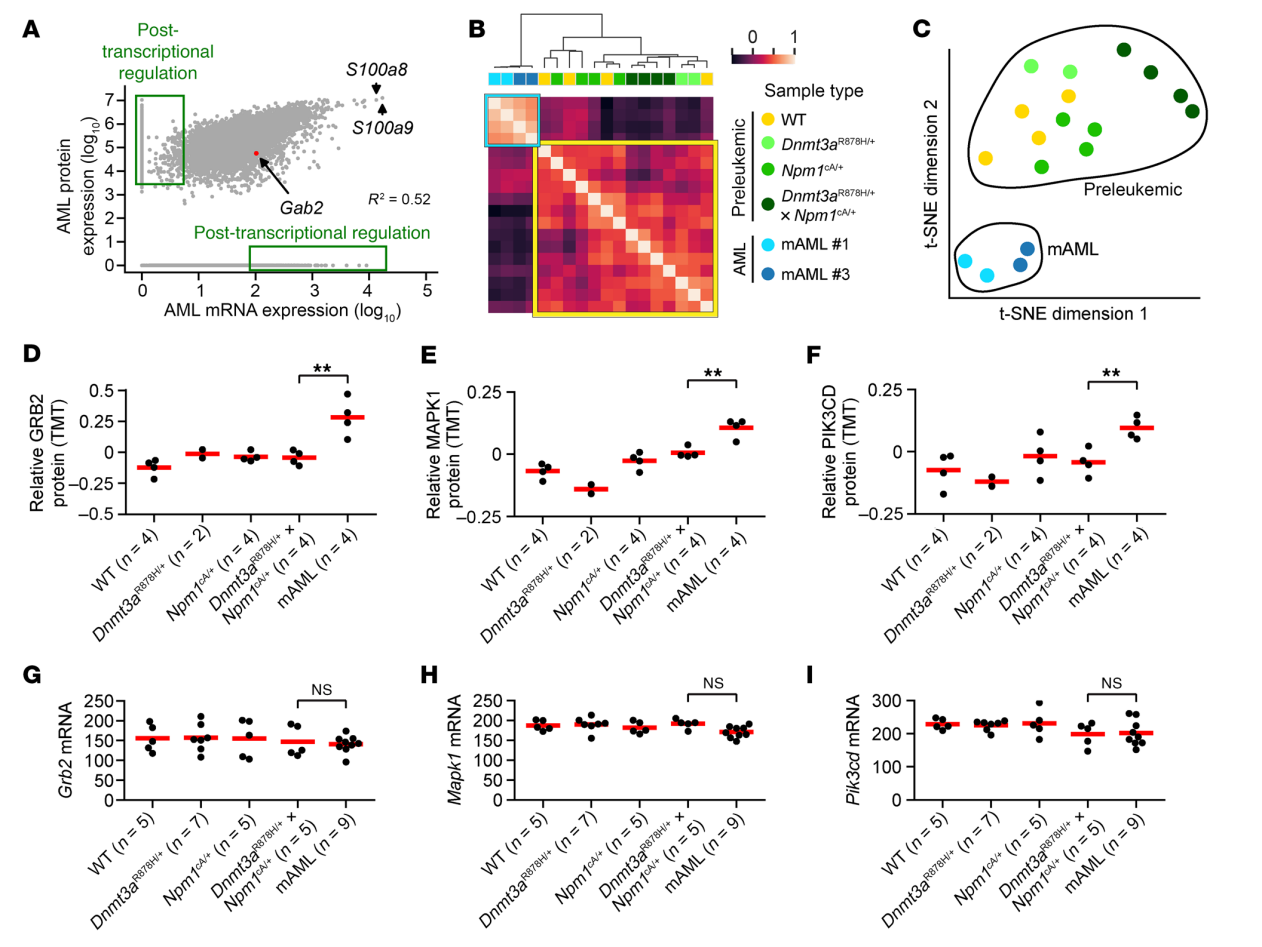
Some RTK/RAS signaling proteins display posttranscriptionally increased protein abundance and NPM1^cA^ physical interactions. (**A**) Mean expression values of protein and mRNA as measured in leukemic mAML murine bone marrow. Protein expression was measured using LFQ mass spectrometry. The *R*^2^ value was calculated on proteins and genes detected using both methods. *S100a9* and *S100a8* are known to be highly abundant genes/proteins in myeloid cells. *Gab2* is indicated in red. (**B**) Hierarchical clustering of TMT proteomics data from preleukemic versus leukemic samples of the indicated genotypes using the unweighted pair group with arithmetic mean (UPGMA) method (76) and Pearson’s correlation of protein abundance profiles as the distance metric. A yellow box highlights preleukemic samples, and a blue box highlights AML samples. (**C**) *t*-SNE created from TMT proteomics data collected from murine bone marrow samples of the indicated types (annotated by color). Preleukemic and leukemic cells formed distinct clusters. mAML samples for **B**–**F** represent 2 biological replicates each of mAML 1 and mAML 3, transplanted into secondary recipients. (**D**–**F**) TMT protein abundance for the indicated genes from bone marrow of preleukemic and leukemic (mAML) mice from this model. Each dot represents a measurement from an individual mouse. TMT protein expression values were median centered at 0 and log_2_ transformed. For mAML samples, adjusted **P < 0.05, by *t* test with Benjamini-Hochberg multiple hypothesis correction for differences compared with *Dnmt3a*^R878H/+^ × *Npm1*^cA/+^ preleukemic bone marrow. Red lines indicate the mean of each group. (**G**–**I**) mRNA expression of the indicated genes from whole bone marrow of preleukemic and leukemic (mAML) mice from this model. NS indicates no significant differential expression in the mAML groups compared with *Dnmt3a*^R878H/+^ × *Npm1*^cA/+^ preleukemic bone marrow. Red lines indicate the mean of each group. Data for PIK3CD are also presented in Supplemental Figure 5C (RNA) and Supplemental Figure 5H (protein).

The RNA-Seq data described above revealed a dramatic transcriptional shift in gene expression patterns associated with transformation, but also suggested that posttranscriptional mechanisms may be relevant for disease pathogenesis. To evaluate posttranscriptional changes in this mouse model, we performed deep-scale tandem-mass-tag (TMT) and label-free quantification (LFQ) mass spectrometry on tryptic peptides from bone marrow samples of preleukemic mice, and also on mAML tumors arising spontaneously in the doubly mutant mice. A total of 9,223 proteins were detected in the TMT dataset, and 7,018 proteins were detected in the LFQ dataset. The measured abundance of most proteins in the AML samples appeared to be regulated by mRNA abundance, with a significant correlation between RNA and LFQ protein abundance measurements (*R^2^* = 0.52; Figure 5A). However, there was also strong evidence for posttranscriptional alterations in protein abundance (designated by green boxes in Figure 5A). Protein abundance measurements clustered the samples into preleukemic and leukemic groups, by both hierarchical clustering of protein abundance measurements and the *t*-distributed stochastic neighbor embedding (*t*-SNE) procedure (42) (Figure 5, B and C), verifying the massive shifts in mRNA expression and protein abundance associated with transformation.

In a previous study by our group on the proteomics of human AML, we observed several proteins with posttranscriptionally increased abundance that also interacted specifically with mutant NPM1^cA^ protein — but not WT NPM1 (43). To extend this to AML cells arising in the mouse model, we identified proteins that met the following criteria: (a) significantly increased abundance in mAML tumors, compared with preleukemic *Dnmt3a*^R878H/+^ *× Npm1*^cA/+^ bone marrow (*P* < 0.05, by 2-sided *t* test adjusted for multiple hypothesis correction by the Benjamini-Hochberg method); (b) no significant change in mRNA expression in mAML tumors, compared with preleukemic *Dnmt3a*^R878H/+^ *× Npm1*^cA/+^ bone marrow; (c) significant physical interactions between NPM1^cA^ and the protein of interest, as measured by TurboID in preleukemic cells; (d) no significant physical interaction between WT NPM1 and the protein of interest, as measured by TurboID in preleukemic cells. Only 20 proteins met all 4 criteria (Supplemental Table 7). The most enriched GO group for these 20 proteins was “intracellular signaling cassette” (FDR < 1 × 10^–7^; 12 of 20 proteins); proteins in this group include GRB2 (the direct interaction partner of GAB2), the signal amplifier MAPK1 (also known as ERK2), and the PI3 kinase p110 subunit PIK3CD (Figure 5, D–I). In our previously published human AML proteomics dataset of 44 patients (43), there were 2 patients with the *DNMT3A*^R882C^ *× NPM1*^c^ genotype who had a markedly higher abundance of both GRB2 and MAPK1 proteins than all the other 42 patients tested, despite an mRNA abundance more comparable to that in other AML samples (data not shown). This observation did not extend to AML samples with *NPM1^c^* mutations and either a non-R882 *DNMT3A* mutation or WT *DNMT3A*. This finding in the mouse model (and possibly in patients) could potentially be caused by stabilization of these proteins via direct NPM1^cA^ interactions, but additional experiments would be required to define the mechanism.

The full RNA-Seq, proteomics, and proximity labeling datasets generated for this study are available for interactive exploration as a community resource at mAML.leylab.org.

### Posttranslational modifications of signaling proteins mediated by GAB2.

**Figure 6:**
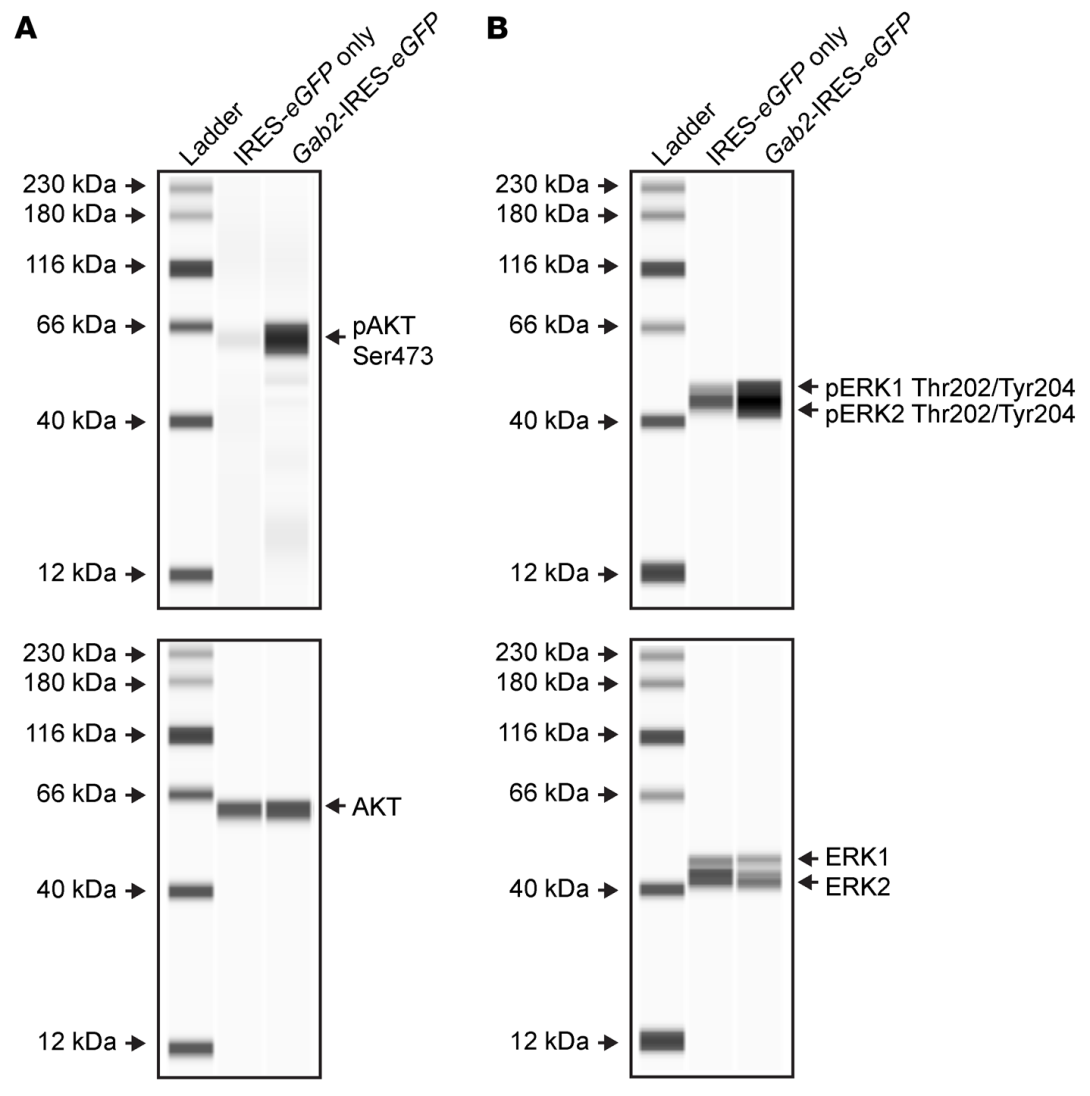
Retroviral overexpression of GAB2 increases phosphorylation of activating sites on AKT and ERK1/2. (**A** and **B**) Preleukemic *Dnmt3a*^R878H/+^ × *Npm1*^cA/+^ bone marrow cells were transduced with MSCV-based retroviruses expressing IRES-*eGFP* only or *Gab2*-IRES-*eGFP* and maintained in vitro until marked selection for *Gab2-*overexpressing cells was observed (>1 month, see Figure 2). GFP^+^ cells were purified and then cultured in serum-reduced media for 24 hours prior to lysis. ProteinSimple Jess Western Blotting performed using anti-phosphorylated AKT (anti-pAKT) (Ser473) (**A**, top), anti-AKT (**A**, bottom), anti-pERK1/2 (Thr202/Tyr204) (**B**, top), or anti-ERK1/2 (**B**, bottom) antibodies. Phosphorylated and total protein blotting was performed on the same blot after stripping using the RePlex system. Note that samples with *Gab2*-IRES-*eGFP* transduction show increased phosphorylation at the activating sites on AKT and ERK1/2 compared with the IRES-*eGFP*–only samples, despite no difference in total AKT or ERK1/2 protein abundance. Both blots are representative of 2 biological and technical replicates.

Given the paucity of transcriptional changes observed with *Gab2* overexpression in preleukemic cells, we hypothesized that *Gab2* overexpression may lead to increased signaling through downstream pathways, such as PI3K/AKT and RAS/RAF/MEK/ERK. To investigate this possibility, we performed Western blotting on preleukemic HSPCs from *Dnmt3a*^R878H/+^ *× Npm1*^cA/+^ mice transduced with *Gab2*-IRES-*eGFP* or IRES-*eGFP* alone, using antibodies against known activation sites on AKT (Ser473) and ERK1/2 (Thr202/Tyr204). Indeed, we observed increased phosphorylation of these activation sites on both AKT and ERK1/2 in the preleukemic *Dnmt3a*^R878H/+^ *× Npm1*^cA/+^ HSPCs transduced with *Gab2*-IRES-*eGFP* compared with those transduced with IRES-*eGFP* alone (Figure 6, A and B). Together, these data suggest that one important effect of *Gab2* overexpression may be increased activation of downstream signaling pathways via AKT, ERK1/2, and/or other relevant pathway proteins.

### Inactivation of Gab2 mitigates the growth of fully transformed murine AML cells.

**Figure 7:**
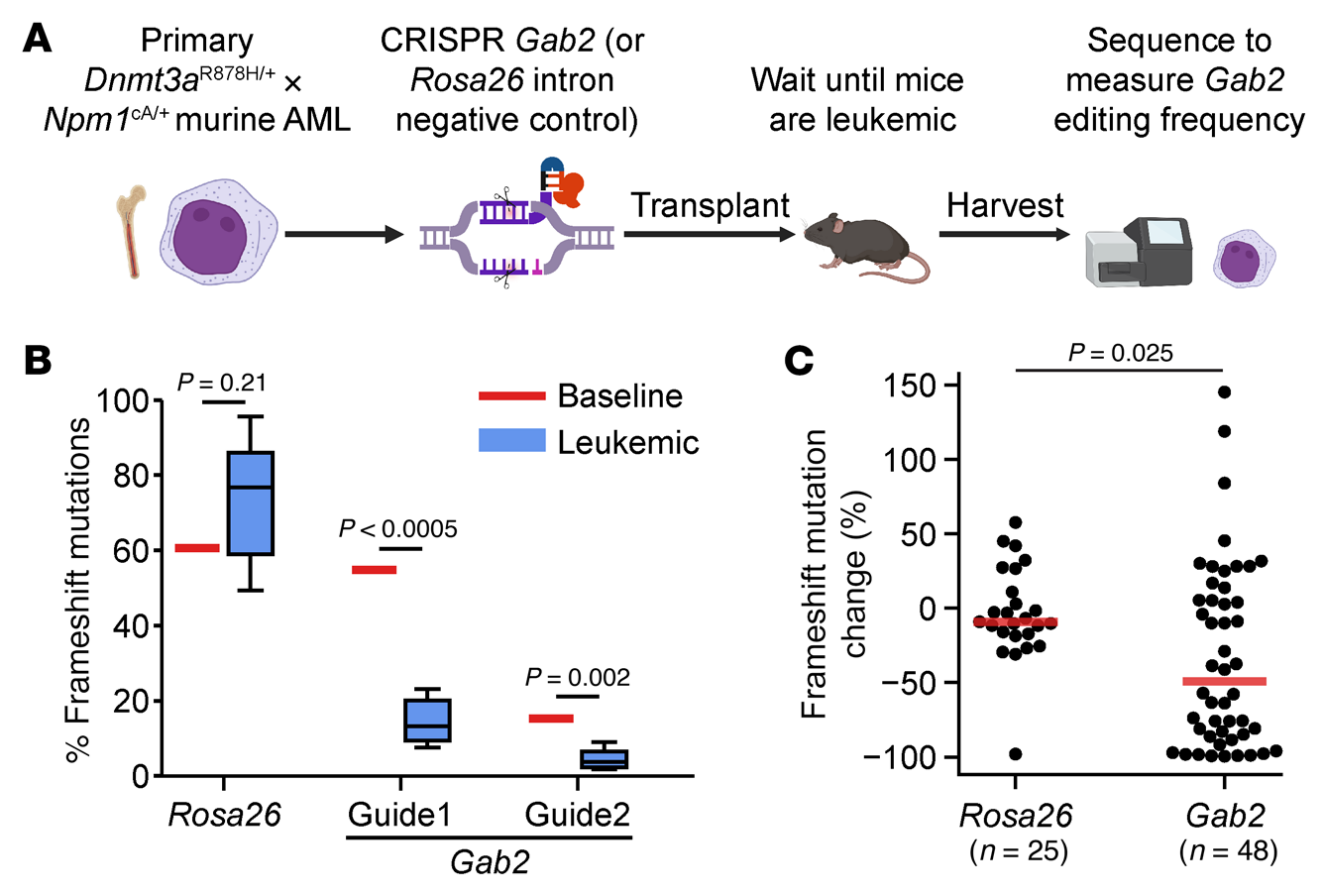
Inactivation of *Gab2* mitigates the growth of some fully transformed mAML cells. (**A**) Schematic of experimental design. *Dnmt3a*^R878H/+^ × *Npm1*^cA/+^ mAML cells were harvested from mouse bone marrow and transfected with Cas9 protein and guide RNA targeting *Gab2* (2 independent guides) or *Rosa26* (negative control). Cells were then transplanted into sublethally irradiated (6 Gy) CD45.1^+^ recipient mice. An aliquot of cells was maintained in vitro for 24–48 hours prior to harvesting to measure baseline editing efficiency. Bone marrow from transplanted mice was harvested when mice were moribund. All mice had more than 90% CD45.2^+^ leukemia cells in the bone marrow at the time of harvesting. DNA from bone marrow was purified, and targeted PCR-based sequencing of edited loci was performed. The figure in **A** was created with BioRender. (**B**) Results from the experiment performed as described in **A**, using mAML 1 (with +7 and *Ptpn11*^E69K^ mutation). *n* = 5 recipient mice per guide. Red line indicates the percentage of frameshift mutations detected at baseline. Boxes show the median (line) and extend from the 25th to 75th IQR, with whiskers showing 1.5 times the IQR; no outlier points were detected beyond the whiskers. *P* values were determined by 2-sided, 1-sample *t* test for differences from the baseline value, adjusted for multiple hypothesis correction using the Benjamini-Hochberg method. (**C**) Results from an experiment performed as described in **A** using 5 independent mAML tumors. Each point represents an individual mouse and shows the percentage of change in frameshift mutations detected at the time of harvesting compared with baseline. Red line indicates the median of each group. *P* values were calculated by Wilcoxon rank-sum test between the *Rosa26* and *Gab2* groups. *Gab2* guides were combined for this plot. See also Supplemental Figure 7 for these data, separated by mAML tumor and individual guide.

To determine whether *Gab2* is required for the maintenance of fully transformed AML cells with +7, we utilized CRISPR/Cas9-mediated genome editing to inactivate *Gab2* (or *Rosa26*, as a negative control) in 5 independent, fully transformed murine AMLs (mAMLs) from this model, and then transplanted these cells into recipients to determine their fate in vivo (Figure 7A). Using a single-guide RNA (sgRNA) to target *Gab2* (or *Rosa26*) creates a semi-random “library” of indels at each targeted site, with variable levels of *Gab2* inactivation achieved in the 3 copies of *Gab2* in each AML cell with +7 (frameshift mutations are the most likely to be deleterious). Digital sequencing studies from the marrow of leukemic mice showed significant selection against frameshift mutations in *Gab2* (using 2 independent guides), but not *Rosa26*, suggesting that inactivation of *Gab2* slowed the growth of fully transformed mAMLs with +7 (Figure 7, B and C). We noted substantial variation from mouse to mouse and tumor to tumor, likely driven by biological differences between individual tumors, as well as variability in the level of *Gab2* inactivation achieved by the library of *Gab2* indels created at baseline in tumors with 3 copies of *Gab2* (e.g., some frameshift mutations in *Gab2* may occur in AML cells that retain 1 or 2 WT copies of *Gab2*) (Supplemental Figure 6).

### Inactivation of GAB2 slows the growth of human AML cells.

**Figure 8:**
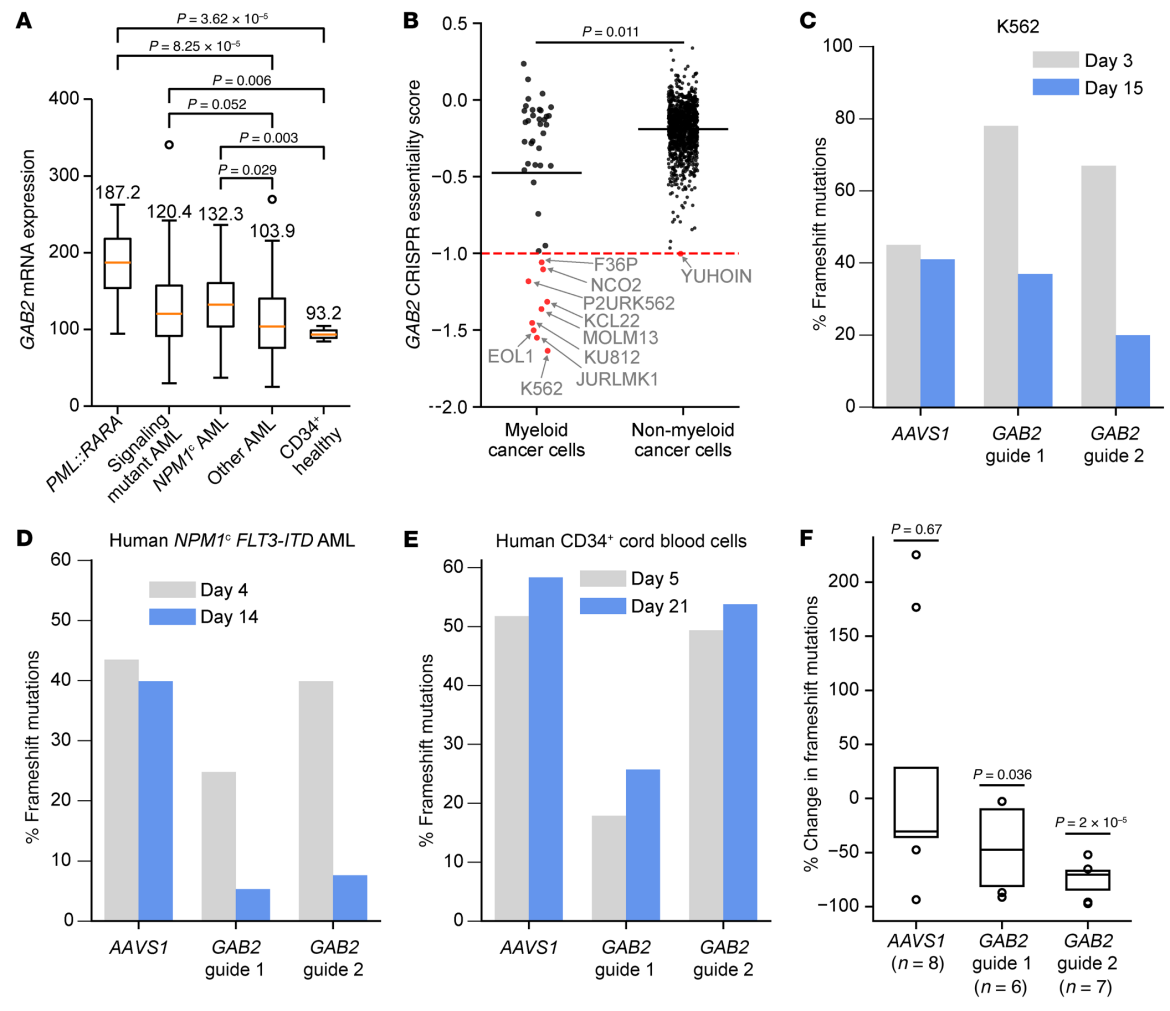
Inactivation of *GAB2* mitigates the growth of some human AML samples. (**A**) *GAB2* mRNA expression in primary human AML samples from TCGA (7), grouped by mutations. *PML::RARA* is the driver of acute promyelocytic leukemia (*n* = 16). Signaling-mutant AML includes 83 patients with *FLT3, NRAS, KRAS, CBL, PTPN11*, or *KIT* mutations; *NPM1*^c^ AML includes 48 patients; other AML includes 72 patients. Purified CD34^+^ cells from healthy donors (CD34^+^ healthy) served as a control (*n* = 3). *P* values were determined by 2-sided, 2-sample *t* test, adjusted with Benjamini-Hochberg multiple hypothesis correction. Boxes show the median (line) and extend from the 25th to 75th IQR, with whiskers 1.5 times the IQR. Numbers indicate the group median. (**B**) GAB2 CRISPR essentiality from the DepMap CHRONOS dataset in human cancer cell lines. Zero (0) indicates no effect. Black line indicates the group mean. Dotted red line shows the median value of pan-essential genes. *P* value was determined by Wilcoxon rank-sum test. (**C**–**E**) Cells were electroporated with *Cas9* mRNA and an sgRNA targeting *GAB2* (2 independent guides) or *AAVS1* (negative control) and maintained in vitro. PCR-based sequencing determined the frequency of frameshift mutations at each targeted site. Cells with frameshift mutations in *GAB2* (but not *AAVS1*) decreased in frequency in (**C**) human K562 cells and (**D**) a cryopreserved, primary human AML sample with *NPM1*^c^ and *FLT3-ITD* mutations, but not in (**E**) CD34^+^ HSPCs from human cord blood (representative of 2 biological replicates). (**F**) Cryopreserved primary human AML samples (each with an *NPM1*^c^ mutation and either *FLT3-ITD*, *FLT3*^D835Y^ or high GAB2 mRNA expression) were CRISPR edited and transplanted into NSG-SGM3 mice. After engraftment, bone marrow was sorted for human AML cells prior to targeted sequencing; sequencing results were compared with baseline editing. *P* values were determined by 2-sided, 1-sample *t* test for differences from 0 in each group. Boxes are as in **A**, with points outside the IQR range shown individually.

We next evaluated the potential relevance of GAB2 for human AML pathogenesis. As noted above, GAB2 mRNA and protein expression levels are higher in human AMLs than in CD34^+^ human cells from healthy donors and/or human promyelocytes from healthy donors (Figure 1, F–H). Consistent with the model that *GAB2* overexpression serves as an intermediate progression event between *NPM1* mutations and downstream RTK/RAS signaling mutations, we observed that *GAB2* expression was significantly higher in human AML with *NPM1* mutations, and/or mutations in signaling genes, compared with other AMLs without these features, and/or healthy donor–derived CD34^+^ cells (Figure 8A). The size of this effect was similar to the approximately 50% increase in GAB2 expression caused by a single-copy gain of chr7 in the mouse AMLs.

To begin to assess the essentiality of GAB2 for human AML, we evaluated available CRISPR-knockout data in human cancer cell lines from the Broad DepMap database (30, 31). These data showed that *GAB2* inactivation markedly reduced the growth of 9 independent human AML cell lines (Figure 8B); we recapitulated these results with 2 *GAB2-*targeted guides (and an *AAVS1* negative control) in K562 cells, which express the BCR::ABL fusion oncoprotein (Figure 8C). Notably, only 1 nonmyeloid human cell line in the DepMap was affected by *GAB2* knockout (YUHOIN melanoma cells), suggesting that GAB2 inactivation may be tolerated in most cells; this is consistent with published results from mouse models, in which germline *Gab2* knockouts are viable, with normal resting blood counts (44, 45). Furthermore, DepMap data for the paralogs *GAB1*, *GAB3*, and *GAB4* did not show dependency in any myeloid cell lines, suggesting that the observed effects may be specific for *GAB2* (Supplemental Figure 7).

We next used CRISPR/Cas9 to inactivate *GAB2* in primary human AML cells with an *NPM1*^c^ founding mutation and a cooperating *FLT3-*ITD signaling mutation. We used 2 independent *GAB2-*targeting guides (and an *AAVS1-*targeting negative control guide) and maintained the cells in vitro with human cytokines (SCF, FLT3L, TPO, IL-3, and IL-6), plated on irradiated human HS-27 stromal cells, as previously described (46). *GAB2* inactivation (but not *AAVS1* targeting) was selected against in these cells (Figure 8D). In contrast, similar experiments with human CD34^+^-selected cord blood samples showed no effect on growth (Figure 8E).

We next performed CRISPR/Cas9 editing in 3 primary human AML samples with *NPM1*^c^ mutations using the same *GAB2* and *AAVS1* guides, and then transplanted these cells into NOD Scid Gamma mice expressing human *SCF,* *GM-CSF,* and *IL-3* (NSG-SGM3 mice) (47). One of these samples had a *FLT3*-ITD cooperating mutation, one had a *FLT3*^D835Y^ mutation, and one was chosen for analysis because of previously identified high *GAB2* mRNA expression. After the mice were engrafted (as measured by peripheral blood flow cytometry), we harvested bone marrow, sorted for human AML cells, and then performed targeted sequencing of the edited loci to determine the fraction of edited cells. We observed selection against *GAB2* frameshift–edited mutations for both *GAB2-*targeting guides, but not for *AAVS1* (Figure 8F). Taken together, these data suggest that *GAB2* inactivation may mitigate the growth of some primary human AML cells.

### GAB2 is transcriptionally activated by the fusion gene PML::RARA in both mouse and human hematopoietic cells.

We noted that human acute promyelocytic leukemia (APL) samples, a subtype of AML initiated by the *PML::RARA* fusion gene (48, 49), had markedly higher expression of *GAB2* than most other AMLs (Figure 8A) (7). APL is also associated with the development of cooperating mutations in signaling genes; in particular, mutations in *FLT3* were seen in 51% of cases (33 of 65) in 1 large cohort (8), similar to the rate of *FLT3* mutations cooperating with *NPM1* (55%; 241 of 436), and significantly higher than in other patients with AML who do not have *NPM1* mutations or *PML::RARA* (22%; 235 of 1,039; *P* < 10^–6^, by χ^2^ test).

**Figure 9:**
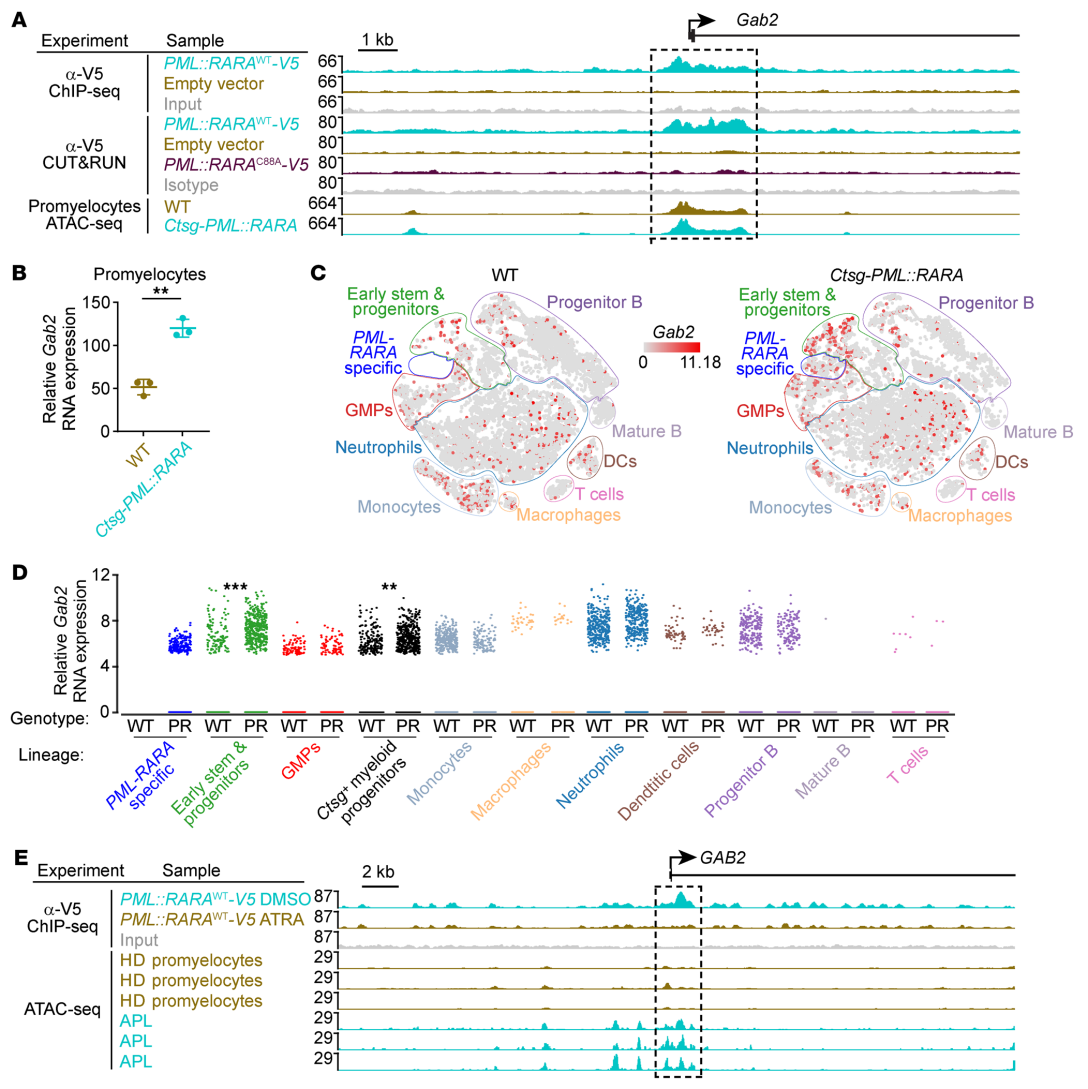
*GAB2* mRNA abundance is increased by *PML::RARA* in both mouse and human hematopoietic cells. (**A**) Genome browser tracks for the *Gab2* locus in mouse for anti-V5 ChIP-Seq or CUT&RUN performed in WT lineage–depleted mouse bone marrow cells transduced with *PML::RARA*^WT^-V5, compared with empty vector–transduced cells or cells transduced with a *PML::RARA*^C88A^-V5 mutant that does not bind to its consensus DNA target sites (50, 77, 78). Assay for transposase-accessible chromatin using sequencing (ATAC-Seq) was performed on promyelocytes that were flow-enriched from the bone marrow of *Ctsg-PML::RARA* mice or WT littermates. (**B**) *Gab2* mRNA expression from bulk RNA-Seq on flow-sorted promyelocytes from three 8- to 12-week-old littermate-matched *Ctsg-PML::RARA* mice versus WT mice. **FDR = 1 × 10^–14^. (**C**) *t*-SNE plots of scRNA-Seq data from whole bone marrow cells from young, nonleukemic WT mice or *Ctsg-PML::RARA* mice (51). Known hematopoietic cell types are labeled according to Haemopedia gene expression profiling (79, 80). A unique population of myeloid precursor cells that were only present in the bone marrow from *Ctsg-PML::RARA* mice is outlined in blue (*PML::RARA* specific). Cells are colored according to *Gab2* expression. GMPs, granulocyte-macrophage progenitors. DCs, dendritic cells. (**D**) Relative *Gab2* expression by scRNA-Seq in the various lineage populations from **C** in WT versus *Ctsg-PML::RARA* (PR) bone marrow. **FDR = 1 × 10^–15^, ***FDR = 1 × 10^–55^. (**E**) Genome browser tracks for the *GAB2* locus in humans for anti-V5 ChIP-Seq performed in healthy donor–derived (HD-derived), human CD34–enriched cord blood cells transduced with *PML::RARA*^WT^-V5, treated with DMSO or ATRA for 48 hours (50); ATRA degrades the PML::RARA protein (52). ATAC-Seq was performed in flow-sorted promyelocytes from healthy donors and in primary human APL samples.

We therefore hypothesized that increased expression of *GAB2* may also play a role in the progression of APL. We examined recently published data from our laboratory investigating the molecular mechanisms of *PML::RARA* action in both murine and human hematopoietic cells (50, 51). In murine lineage-depleted primary bone marrow cells, we found that PML::RARA directly bound to a region near the 5′ end of *Gab2*, defined by both ChIP-Seq and CUT&RUN; this region had accessible chromatin by ATAC-Seq in WT promyelocytes (Figure 9A). No binding at this region was observed using a mutant PML::RARA^C88A^ protein that does not bind its consensus DNA targets, as a negative control. In RNA-Seq data from mice expressing *PML::RARA* under the control of a promyelocyte-specific cathepsin G (*Ctsg*) promoter, we observed a 2.32-fold increase in *Gab2* expression in promyelocytes compared with expression in WT promyelocytes (FDR < 1 × 10^–14^; Figure 9B). In single-cell RNA-Seq (scRNA-Seq) data, we observed that the increase in *Gab2* expression primarily occurred in early stem and progenitor cells, in a unique population of cells that is found only in samples with *PML::RARA* expression, and in *Ctsg*-expressing myeloid progenitors (Figure 9, C and D).

We observed a similar phenomenon in human hematopoietic cells. In human CD34^+^ cells from cord blood, we observed PML::RARA binding near the 5′ end of *GAB2*, using an anti-V5 antibody to perform ChIP-Seq on V5-tagged PML::RARA (Figure 9E). This binding peak occurred in a region with accessible chromatin found only in APL cells, but not in healthy donor–derived promyelocytes. Treatment of these cells with all-trans retinoic acid (ATRA), a compound that induces degradation of the PML::RARA protein (52, 53), eliminated binding to this *GAB2*-adjacent peak (Figure 9E). Previously published scRNA-Seq data from our group (50) demonstrated a 2.64-fold increase in *GAB2* expression in human CD34^+^ cord blood cells transfected with *PML::RARA* compared with those transfected with a mutant *PML::RARA*^C88A^ construct that does not bind to its consensus DNA target sequence (FDR < 1 × 10^–10^). Finally, 20 genes have an identified PML::RARA binding site within 1 kb of their transcription start sites in both mouse and human hematopoietic cells and display increased expression in *PML::RARA*-expressing promyelocytes: *GAB2* is one of these genes (Supplemental Table 8).

Taken together, these data suggest that PML::RARA may directly bind to the 5′ flanking region of *GAB2* and activate its transcription, leading to an increase in *GAB2* expression in APL cells. This suggests at least 1 alternative mechanism for *GAB2* overexpression that does not rely on amplification of this gene.

### Gab2 inactivation mitigates the growth of murine APL cells.

**Figure 10:**
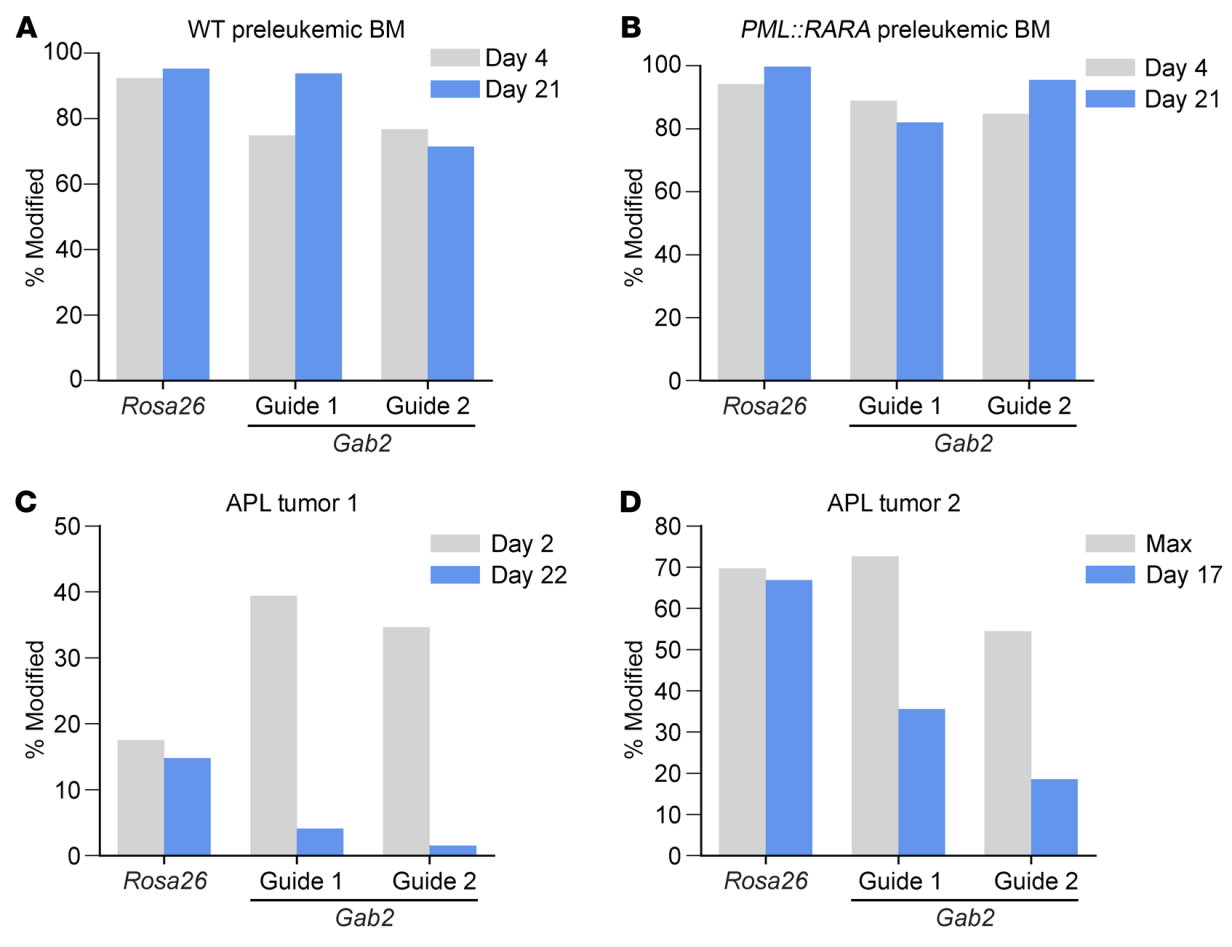
*Gab2* inactivation mitigates the growth of murine APL cells. (**A** and **B**) Bone marrow cells from WT (**A**) or *Ctsg-PML::RARA* (**B**) mice were harvested and electroporated with Cas9 protein and sgRNAs targeting *Gab2* (2 independent guides) or *Rosa26* (as a negative control). Cells were cultured in methylcellulose and replated weekly. Targeted PCR-based sequencing was performed at the indicated time points to determine the fraction of edited cells. (**C** and **D**) Cryovials of cells from 2 independent murine APLs were thawed and transfected with *Cas9* mRNA and an sgRNA targeting *Gab2* (2 independent guides) or *Rosa26* (as a negative control). Cells were cultured in methylcellulose and replated every 5–7 days. Targeted PCR-based sequencing was performed at the indicated time points to determine the fraction of edited cells. For APL tumor 2 (**D**), editing efficiency was maximal (Max) at day 3 for *Rosa26* and *Gab2* guide 1, and at day 10 for *Gab2* guide 2 (other time points were not measured). The maximum value is displayed for each guide for baseline comparison.

We performed CRISPR/Cas9 editing of WT murine bone marrow, preleukemic bone marrow from *Ctsg*-*PML::RARA* mice, or fully transformed murine APLs from this model (54) using guides targeting *Gab2* (or *Rosa26* as a negative control), and then maintained these cells in vitro in methycellulose media (MethoCult M3434) for approximately 3 weeks (51, 55). We observed minimal selection against edited cells in the preleukemic setting (with or without *PML::RARA*; Figure 10, A and B). However, we did observe marked selection against edited cells for the *Gab2* guides (but not *Rosa26* guides) in 2 independent murine APL tumors arising spontaneously in this *Ctsg*-*PML::RARA* model (Figure 10, C and D). These data suggest that, while *Gab2* transcription was directly increased by PML::RARA, the cells became dependent on *Gab2* only after they were fully transformed.

## Discussion

Genome sequencing studies of human AML samples have identified a significant association between initiating mutations (e.g., *NPM1*, *PML::RARA*) and the development of downstream cooperating mutations in RTK/RAS signaling genes. In this study, we were able to identify an intermediate step in AML transformation — the amplification of mouse chr7 — acquired between the initiating events and the downstream RTK/RAS signaling mutations. The identification of +7 (without associated RTK/RAS mutations) in nontransplantable clonal expansions was critical for identifying +7 as the probable third hit in this model, occurring prior to the development of RTK/RAS signaling mutations. This observation, along with other lines of evidence, nominated the *Gab2* and *Pak1* genes within the recurrently amplified 8.9 Mbp region on chr7 as candidates for functional validation. Using retroviral constructs, we found that overexpression of *Gab2* (but not *Pak1*) induced significant (*P* < 1 × 10^–10^ by 2-sided, 1-sample *t* test at 10 weeks) expansion in hematopoietic cells with *Npm1*^cA^ and *Dnmt3a*^R878H^ mutations and accelerated the development of AML; these effects were dependent on the preexisting mutations in *Dnmt3a* and *Npm1*. These studies led to the identification of *Gab2* as a potentially relevant gene for AML pathogenesis. However, the amplification of chr7 observed in some GAB2-overexpressing mAMLs suggests that 1 or more additional genes on chr7 may also be relevant for AML pathogenesis. Additional studies will be required to identify this gene or genes.

Our studies have suggested that *Gab2* overexpression is tolerated only in the presence of the *Npm1*^cA^ mutation and that *Gab2* overexpression, once established, facilitates the process of AML transformation. In the mouse model used in this study, *Gab2* overexpression was achieved primarily through chromosomal amplification. However, in human AML samples, the region of chromosome 11q14.1 containing *GAB2* was amplified in only 1%–2% of cases (8, 56); data from clinical whole-genome sequencing performed on patients with AML at our institution identified amplification in 3.1% of cases (14 of 445), primarily in patients with *TP53* mutations and/or complex karyotypes (57). Increased *GAB2* expression, however, is substantially more common in AML (35% of AML patient samples from The Cancer Genome Atlas [TCGA] AML study have *GAB2* mRNA expression >1.5-fold higher than that of CD34^+^ cells) and is associated with *NPM1* mutations, RTK/RAS signaling mutations, and *PML::RARA* fusions. We identified binding of the *GAB2* 5′ flanking region by the PML::RARA fusion protein as a transcriptional mechanism that may directly increase *GAB2* expression; the *PML::RARA* fusion is also commonly associated with cooperating mutations that activate signaling (7, 8). The mechanisms that cause increased *GAB2* expression in patients with AML without *PML::RARA* or 11q14.1 amplification remain to be determined.

In other malignancies, recurrent amplification of 11q14.1 (containing *GAB2*) has been identified in breast cancer (24.7% of patients), ovarian cancer (44% of patients), and acral melanoma (47% of patients) (58–61). In ovarian cancer, systematic evaluation of candidate genes from this region identified *GAB2* as the likely oncogenic driver (58). In acral melanoma with an amplified 11q13-14 region, investigators were able to identify the presence of 11q13-14 amplification in nearby melanoma in situ lesions in almost every case, suggesting that *GAB2* overexpression may also play an early, facilitating role in these tumors. Strikingly, the study of acral melanoma also identified recurrent driver mutations in RTK/RAS signaling genes, including *PTPN11*, *KIT*, *NF1*, *KRAS*, and *NRAS*, all of which are recurrent in human AML (and commonly associated with *NPM1*^c^ mutations). These observations suggest that *GAB2* overexpression may facilitate downstream RTK/RAS signaling mutations in other malignancies as well.

How does *GAB2* overexpression facilitate AML progression? It does not appear to directly cause substantial transcriptional changes in preleukemic murine bone marrow cells with *Dnmt3a* and *Npm1* mutations, but several lines of evidence suggest it may increase signaling through posttranscriptional and posttranslational mechanisms, including the activation of downstream signaling molecules such as AKT and ERK. GAB2 is known to act as a coordinator and transmitter of signals; it physically interacts with GRB2, PTPN11, and CBL to transmit signals from RTKs (including FLT3 and KIT) to downstream pathways (including PI3K, AKT, and RAS, in some contexts) (62). The paucity of additional RTK/RAS mutations in GAB2 mAML suggests that, if sufficiently overexpressed, *GAB2* can independently facilitate the activated growth signaling required for transformation. Cells with approximately 50% increased *GAB2*, on the other hand, may be “primed” to amplify oncogenic signaling from acquired mutations in RTK/RAS genes. This hypothesis is supported by studies suggesting that knockout of *GAB2* can reduce or eliminate the ability of *BCR::ABL* (63–65), mutant *FLT3* (66, 67), and/or mutant *PTPN11* (68, 69) to transform cells. Additional studies will be required to determine how *GAB2* amplifies signals generated by these oncogenic drivers.

Most mutant NPM1^c^ is mislocalized to the cytoplasm in AML cells. While a role for NPM1^c^ in the nucleus has been suggested (22, 23), the function or functions of cytoplasmic NPM1^c^ remain unclear. Here, we identify the physical proximity of mutant NPM1^cA^ with GAB2 and its partner, GRB2. Combined with the observation that tolerance of *Gab2* overexpression is dependent on the presence of mutant NPM1^cA^, these studies suggest that its physical interaction with some of these proteins in AML cells may be relevant for the mechanism of NPM1^c^ mutations, perhaps by protecting them from degradation. The observation that GAB2 and NPM1^cA^ interact only in *Dnmt3a*^R878H/+^ cells, but not in WT cells, suggests a molecular mechanism that may be relevant for the synergy generated by *DNMT3A* and *NPM1* mutations.

Our studies also suggest that GAB2 inhibition may represent a therapeutic target for human AML, since some human AML cell lines and primary human AML cells are susceptible to CRISPR inactivation of *GAB2*. If effective, this approach could have applicability in patients with a variety of mutations that signal through GAB2. Furthermore, HSPCs from healthy individuals did not show altered growth with CRISPR inactivation of *GAB2*, and publicly available CRISPR screening data from the Broad DepMap did not show an effect of *GAB2* inactivation in nonmyeloid cells. Mice with germline *Gab2* inactivation are known to be viable, fertile, and display normal resting blood counts (44, 45). Mechanistically, this may be due to redundancy across the *GAB* family member genes (*Gab1*, *Gab2*, *Gab3*, and *Gab4*), with previous studies suggesting that *Gab1* (the most ubiquitously expressed family member) may be redundant with *Gab2* in cardiac muscle, while *Gab3* appears to have redundant function with *Gab2* in immune cells (59, 70–72); regardless, only *GAB2* inactivation shows a deleterious effect in AML cell lines. While GAB2 inactivation can slow the growth of some AML cells, the effect is not ubiquitous; further work will be needed to determine the factors that govern susceptibility. Recent studies have identified small molecules that can disrupt the binding of GAB2 to GRB2 (73, 74); additional studies will be required to determine whether blocking the interactions of GAB2 with its downstream partners, targeted GAB2 protein degradation, the inhibition of pathways downstream of GAB2, and/or other GAB2-targeting strategies may represent potential approaches for a variety of malignancies, including AML.

Taken together, this study advances our understanding of AML (and other malignancies) in several ways: (a) it provides a mechanistic missing link between initiating events in AML and RTK/RAS signaling mutations; (b) it strengthens the body of evidence for GAB2 as a relevant target by demonstrating GAB2 dependency of some fully transformed, primary AML samples; (c) it provides evidence that cytoplasmic NPM1^cA^ may play a role in downstream signaling events, by virtue of a direct interaction with GAB2 and/or other signaling proteins; and (d) it helps to establish a recurrent pattern of GAB2 overexpression prior to the development of RTK/RAS signaling mutations in a variety of cancers, suggesting that GAB2 may represent a broadly relevant therapeutic target. Additional studies will be required to better define the synergistic interactions of DNMT3A^R882H^ and NPM1^cA^ with GAB2 and the potential therapeutic value of blocking these interactions.

## Methods

### Sex as a biological variable.

Our study examined male and female animals and human patient samples, and similar findings are reported for both sexes.

Additional details can be found in the Supplemental Methods.

### Statistics.

DEGs in RNA-Seq data were called using the edgeR package, with FDR cutoffs as indicated. Enrichment for Gene Ontology terms was calculated using the hypergeometric test, corrected for multiple hypothesis testing using the Benjamini-Hochberg method. Other statistical testing was performed using the 2-tailed *t* test, Wilcoxon rank-sum test, χ^2^ or log-rank test, with multiple hypothesis correction by the Benjamini-Hochberg method. *P* values of less than 0.05 were considered significant. Data are presented as median ± IQR or mean ± SD, as indicated in figure legends.

### Study approval.

Human samples were acquired as part of studies approved by the Human Research Protection Office at Washington University. All patients provided written informed consent prior to participation in the study under IRB protocol no. 201011766 or no. 202104011. Murine studies were approved under IACUC protocol 23-0041, Washington University School of Medicine.

### Data availability.

RNA-Seq, mass spectrometry, and TurboID data are available for exploration and can be downloaded on the interactive website mAML.leylab.org. Mass spectrometric data is deposited in the MassIVE database (MSV000097966 [TurboID], MSV000098719 [TMT], MSV000098728 [LFQ]). Genomic and transcriptomics sequencing data have been deposited in the NCBI’s Sequence Read Archive (SRA) under accession code PRJNA1261866. Plasmids created for this study have been deposited in Addgene (plasmid nos. 240126 through 240134). Values for all data points in graphs are reported in the Supporting Data Values file.

## Author contributions

MHK and TJL designed research studies. MHK, SNR, YL, TY, NMH, DRG, MC, and CDSK conducted experiments and acquired data. MHK, CDSK, SMR, and CAM analyzed data. MHK, CAM, and TJL wrote the manuscript.

## Funding support

This work is the result of NIH funding, in whole or in part, and is subject to the NIH Public Access Policy. Through acceptance of this federal funding, the NIH has been given a right to make the work publicly available in PubMed Central.

- National Cancer Institute (NCI)/NIH SPORE in Leukemia P50CA171963 (Daniel Link, PI), Career Enhancement Program (CEP) award (to MHK).
- American Society of Hematology (ASH) Fellow Scholar Award (to MHK).
- NCI grants P01CA101937, R35CA197561 (to TJL).
- NCI grant K22CA272967 (to CDSK).
- Barnes Jewish Hospital Foundation Award (to TJL).
- ASH Fellow to Faculty Scholar Award (to CDSK).
- NCI grant R50CA211782 (to CAM).
- Washington University Institute of Clinical and Translational Sciences (National Center for Advancing Translational Sciences [NCATS], NIH grant UL1 TR000448).
- Mass Spectrometry Research Resource (National Institute of General Medical Sciences, [NIGMS], NIH grants P41GM103422 and R24GM136766).
- Siteman Comprehensive Cancer Center Support Grant (NCI grant P30CA091842).

## Supplementary Material

Supplemental data

Unedited blot and gel images

Supplemental table 1

Supplemental table 2

Supplemental table 3

Supplemental table 4

Supplemental table 5

Supplemental table 6

Supplemental table 7

Supplemental table 8

Supporting data values

## Version 1 08/07/2025

In-Press Preview

## Version 2 11/03/2025

Electronic publication

## References

[1] Shallis RM (2019). Epidemiology of acute myeloid leukemia: recent progress and enduring challenges. Blood Rev.

[2] https://seer.cancer.gov/statfacts/html/amyl.html.

[3] Welch JS (2012). The origin and evolution of mutations in acute myeloid leukemia. Cell.

[4] Xie M (2014). Age-related mutations associated with clonal hematopoietic expansion and malignancies. Nat Med.

[5] Jaiswal S (2014). Age-related clonal hematopoiesis associated with adverse outcomes. N Engl J Med.

[6] Bowman RL (2018). Clonal hematopoiesis and evolution to hematopoietic malignancies. Cell Stem Cell.

[7] Ley TJ (2013). Genomic and epigenomic landscapes of adult de novo acute myeloid leukemia. N Engl J Med.

[8] Papaemmanuil E (2016). Genomic classification and prognosis in acute myeloid leukemia. N Engl J Med.

[9] Ley TJ (2010). DNMT3A mutations in acute myeloid leukemia. N Engl J Med.

[10] Klco JM (2014). Functional heterogeneity of genetically defined subclones in acute myeloid leukemia. Cancer Cell.

[11] Okano M (1999). DNA methyltransferases Dnmt3a and Dnmt3b are essential for de novo methylation and mammalian development. Cell.

[12] Smith AM (2022). Somatic Dnmt3a inactivation leads to slow, canonical DNA methylation loss in murine hematopoietic cells. iScience.

[13] Russler-Germain DA (2014). The R882H DNMT3A mutation associated with AML dominantly inhibits WT DNMT3A by blocking its ability to form active tetramers. Cancer Cell.

[14] Spencer DH (2017). CpG island hypermethylation mediated by DNMT3A is a consequence of AML progression. Cell.

[15] Riback JA (2020). Composition-dependent thermodynamics of intracellular phase separation. Nature.

[16] Falini B (2006). Both carboxy-terminus NES motif and mutated tryptophan(s) are crucial for aberrant nuclear export of nucleophosmin leukemic mutants in NPMc+ AML. Blood.

[17] Grummitt CG (2008). Structural consequences of nucleophosmin mutations in acute myeloid leukemia. J Biol Chem.

[18] Falini B (2005). Cytoplasmic nucleophosmin in acute myelogenous leukemia with a normal karyotype. N Engl J Med.

[19] Bolli N (2007). Born to be exported: COOH-terminal nuclear export signals of different strength ensure cytoplasmic accumulation of nucleophosmin leukemic mutants. Cancer Res.

[20] Falini B (2020). NPM1-mutated acute myeloid leukemia: from bench to bedside. Blood.

[21] Brunetti L (2018). Mutant NPM1 maintains the leukemic state through HOX expression. Cancer Cell.

[22] Uckelmann HJ (2023). Mutant NPM1 directly regulates oncogenic transcription in acute myeloid leukemia. Cancer Discov.

[23] Wang XQD (2023). Mutant NPM1 hijacks transcriptional hubs to maintain pathogenic gene programs in acute myeloid leukemia. Cancer Discov.

[24] Loberg MA (2019). Sequentially inducible mouse models reveal that Npm1 mutation causes malignant transformation of Dnmt3a-mutant clonal hematopoiesis. Leukemia.

[25] SanMiguel JM (2022). Cell origin-dependent cooperativity of mutant Dnmt3a and Npm1 in clonal hematopoiesis and myeloid malignancy. Blood Adv.

[26] Guryanova OA (2016). DNMT3A mutations promote anthracycline resistance in acute myeloid leukemia via impaired nucleosome remodeling. Nat Med.

[27] Lao Z (2012). MASTR: a technique for mosaic mutant analysis with spatial and temporal control of recombination using conditional floxed alleles in mice. Cell Rep.

[28] Dovey OM (2017). Molecular synergy underlies the co-occurrence patterns and phenotype of NPM1-mutant acute myeloid leukemia. Blood.

[29] Suehnholz SP (2024). Quantifying the expanding landscape of clinical actionability for patients with cancer. Cancer Discov.

[30] https://plus.figshare.com/articles/dataset/DepMap_23Q4_Public/24667905/2.

[31] Tsherniak A (2017). Defining a cancer dependency map. Cell.

[32] Luo Q (2024). Targetable leukaemia dependency on noncanonical PI3Kγ signalling. Nature.

[33] Pandolfi A (2015). PAK1 is a therapeutic target in acute myeloid leukemia and myelodysplastic syndrome. Blood.

[34] Suzuki T (2002). New genes involved in cancer identified by retroviral tagging. Nat Genet.

[35] Bedigian HG (1984). Spontaneous and induced leukemias of myeloid origin in recombinant inbred BXH mice. J Virol.

[36] Li J (1999). Leukaemia disease genes: large-scale cloning and pathway predictions. Nat Genet.

[37] Nakamura T (1996). Cooperative activation of Hoxa and Pbx1-related genes in murine myeloid leukaemias. Nat Genet.

[38] Smith AM (2021). Functional and epigenetic phenotypes of humans and mice with DNMT3A overgrowth syndrome. Nat Commun.

[40] Becht E Dimensionality reduction for visualizing single-cell data using UMAP. Nat Biotechnol.

[41] Branon TC (2018). Efficient proximity labeling in living cells and organisms with TurboID. Nat Biotechnol.

[42] Van der Maaten L, Hinton G (2008). Visualizing data using t-SNE. J Mach Learn Res.

[43] Kramer MH (2022). Proteomic and phosphoproteomic landscapes of acute myeloid leukemia. Blood.

[44] Zhang Y (2007). Abnormal hematopoiesis in Gab2 mutant mice. Blood.

[45] Gu H (2001). Essential role for Gab2 in the allergic response. Nature.

[46] Klco JM (2013). Genomic impact of transient low-dose decitabine treatment on primary AML cells. Blood.

[47] Wunderlich M (2010). AML xenograft efficiency is significantly improved in NOD/SCID-IL2RG mice constitutively expressing human SCF, GM-CSF and IL-3. Leukemia.

[48] Khoury JD (2022). The 5th edition of the World Health Organization classification of haematolymphoid tumours: myeloid and histiocytic/dendritic neoplasms. Leukemia.

[49] Arber DA (2022). International Consensus Classification of myeloid neoplasms and acute leukemias: integrating morphologic, clinical, and genomic data. Blood.

[50] Katerndahl CDS (2024). PML::RARA and GATA2 proteins interact via DNA templates to induce aberrant self-renewal in mouse and human hematopoietic cells. Proc Natl Acad Sci U S A.

[51] Katerndahl CDS (2021). Tumor suppressor function of Gata2 in acute promyelocytic leukemia. Blood.

[52] Zhu J (1999). Retinoic acid induces proteasome-dependent degradation of retinoic acid receptor alpha (RARalpha) and oncogenic RARalpha fusion proteins. Proc Natl Acad Sci U S A.

[53] Lo-Coco F (2013). Retinoic acid and arsenic trioxide for acute promyelocytic leukemia. N Engl J Med.

[54] Westervelt P (2003). High-penetrance mouse model of acute promyelocytic leukemia with very low levels of PML::RARAlpha expression. Blood.

[55] Cole CB (2016). PML::RARA requires DNA methyltransferase 3A to initiate acute promyelocytic leukemia. J Clin Invest.

[56] Zatkova A (2006). GAB2 is a novel target of 11q amplification in AML/MDS. Genes Chromosomes Cancer.

[57] Duncavage EJ (2021). Genome sequencing as an alternative to cytogenetic analysis in myeloid cancers. N Engl J Med.

[58] Dunn GP (2014). In vivo multiplexed interrogation of amplified genes identifies GAB2 as an ovarian cancer oncogene. Proc Natl Acad Sci U S A.

[59] Wang M (2024). The genetic evolution of acral melanoma. Nat Commun.

[60] Bastian BC (2000). Gene amplifications characterize acral melanoma and permit the detection of occult tumor cells in the surrounding skin. Cancer Res.

[61] Bocanegra M (2010). Focal amplification and oncogene dependency of GAB2 in breast cancer. Oncogene.

[62] Ding CB (2015). Structure and function of Gab2 and its role in cancer (Review). Mol Med Rep.

[63] Halbach S (2016). Gab2 is essential for Bcr-Abl-mediated leukemic transformation and hydronephrosis in a chronic myeloid leukemia mouse model. Leukemia.

[64] Gu S (2016). Distinct GAB2 signaling pathways are essential for myeloid and lymphoid transformation and leukemogenesis by BCR-ABL1. Blood.

[65] Sattler M (2002). Critical role for Gab2 in transformation by BCR/ABL. Cancer Cell.

[66] Spohr C (2022). Gab2 deficiency prevents Flt3-ITD driven acute myeloid leukemia in vivo. Leukemia.

[67] Sies K (2019). Gab2 is essential for transformation by FLT3-ITD in acute myeloid leukemia. Hemasphere.

[68] Mohi MG (2005). Prognostic, therapeutic, and mechanistic implications of a mouse model of leukemia evoked by Shp2 (PTPN11) mutations. Cancer Cell.

[69] Liu W (2017). Inhibition of the Gab2/PI3K/mTOR signaling ameliorates myeloid malignancy caused by Ptpn11 (Shp2) gain-of-function mutations. Leukemia.

[70] Nakaoka Y (2007). Gab family proteins are essential for postnatal maintenance of cardiac function via neuregulin-1/ErbB signaling. J Clin Invest.

[71] Itoh M (2000). Role of Gab1 in heart, placenta, and skin development and growth factor- and cytokine-induced extracellular signal-regulated kinase mitogen-activated protein kinase activation. Mol Cell Biol.

[72] Sachs M (2000). Essential role of Gab1 for signaling by the c-Met receptor in vivo. J Cell Biol.

[73] Bufano M (2023). Targeting the Grb2 cSH3 domain: design, synthesis and biological evaluation of the first series of modulators. Bioorg Chem.

[74] Malagrino F (2020). Targeting the interaction between the SH3 domain of Grb2 and Gab2. Cells.

[75] Tyner JW (2018). Functional genomic landscape of acute myeloid leukaemia. Nature.

[76] https://ia800201.us.archive.org/13/items/cbarchive_33927_astatisticalmethodforevaluatin1902/astatisticalmethodforevaluatin1902.pdf.

[77] Liu X (2014). The DNA binding property of PML/RARA but not the integrity of PML nuclear bodies is indispensable for leukemic transformation. PLoS One.

[78] Durand B (1994). Activation function 2 (AF-2) of retinoic acid receptor and 9-cis retinoic acid receptor: presence of a conserved autonomous constitutive activating domain and influence of the nature of the response element on AF-2 activity. EMBO J.

[79] Ketkar S (2020). Remethylation of Dnmt3a^–/–^ hematopoietic cells is associated with partial correction of gene dysregulation and reduced myeloid skewing. Proc Natl Acad Sci U S A.

[80] Petti AA (2019). A general approach for detecting expressed mutations in AML cells using single cell RNA-sequencing. Nat Commun.

