# Supplementary material for: Overexpression of the signaling coordinator GAB2 can play an important role in acute myeloid leukemia progression
Source: J Clin Invest. 2025 Aug 7;135(21):e195929. doi: 10.1172/JCI195929 (PMC12578389; doi:10.1172/JCI195929)
Supplement: Supplemental data [file jci-135-195929-s150.pdf]

SUPPLEMENTAL MATERIALS

Table of Contents 1
Supplemental Figure 1........ 2
Supplemental Figure 2........ 3
Supplemental Figure 3........ 4
Supplemental Figure 4........ 5
Supplemental Figure 5........ 6
Supplemental Figure 6........ 7
Supplemental Figure 7........ 8
Captions for Supplemental Tables........ 9
Supplemental Methods........ 10
Supplemental References 14

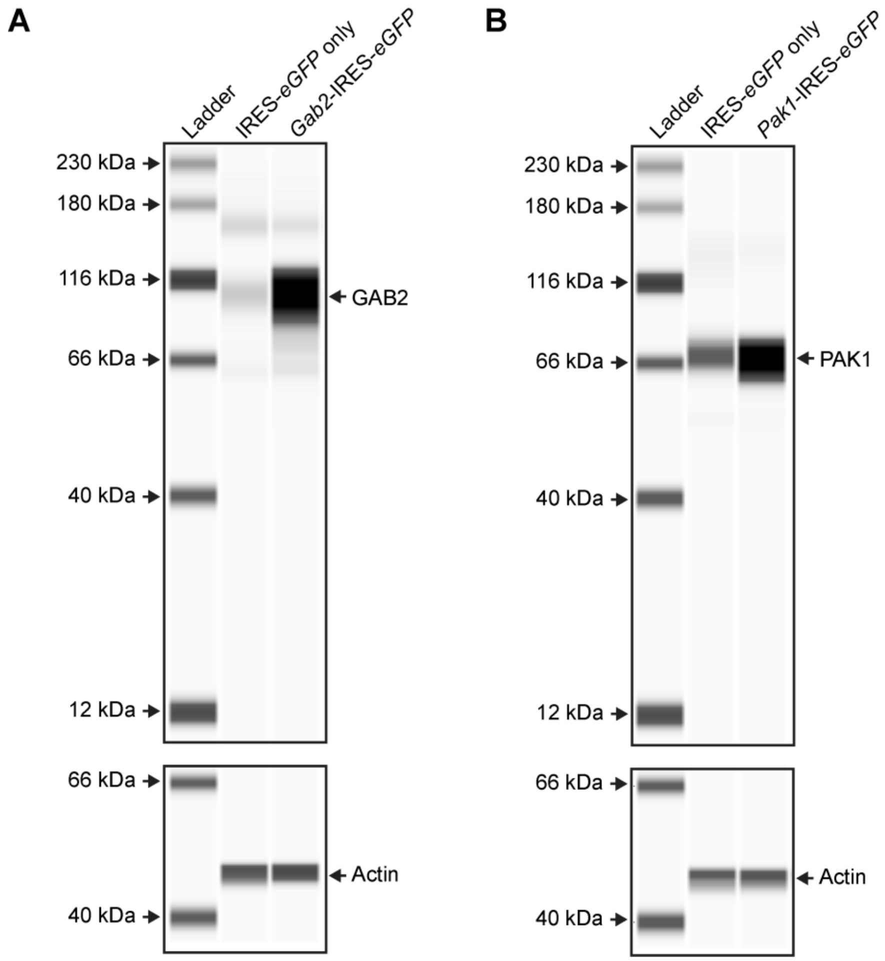

Supplemental Figure 1. Retroviral overexpression of GAB2 and PAK1 proteins confirmed by Western blotting. (A, B) Preleukemic murine *Dnmt3a*$^{R878H/+}$ x *Npm1*$^{cA/+}$ bone marrow cells were transduced with MSCV-based retroviruses with IRES-*eGFP* only, *Gab2*-IRES-*eGFP*, or *Pak1*-IRES-*eGFP*. GFP+ cells were purified and lysed at one week. ProteinSimple Jess Western Blotting was performed using anti-GAB2 (Panel A) or anti-PAK1 (Panel B) antibodies. The loading control with anti-Actin antibodies was performed on the same blot after stripping, using the RePlex system. Note that GAB2 has a molecular weight of 74.5 kDa, but an apparent molecular weight of 90-100 kDa on Western blot, as previously reported (1-3).

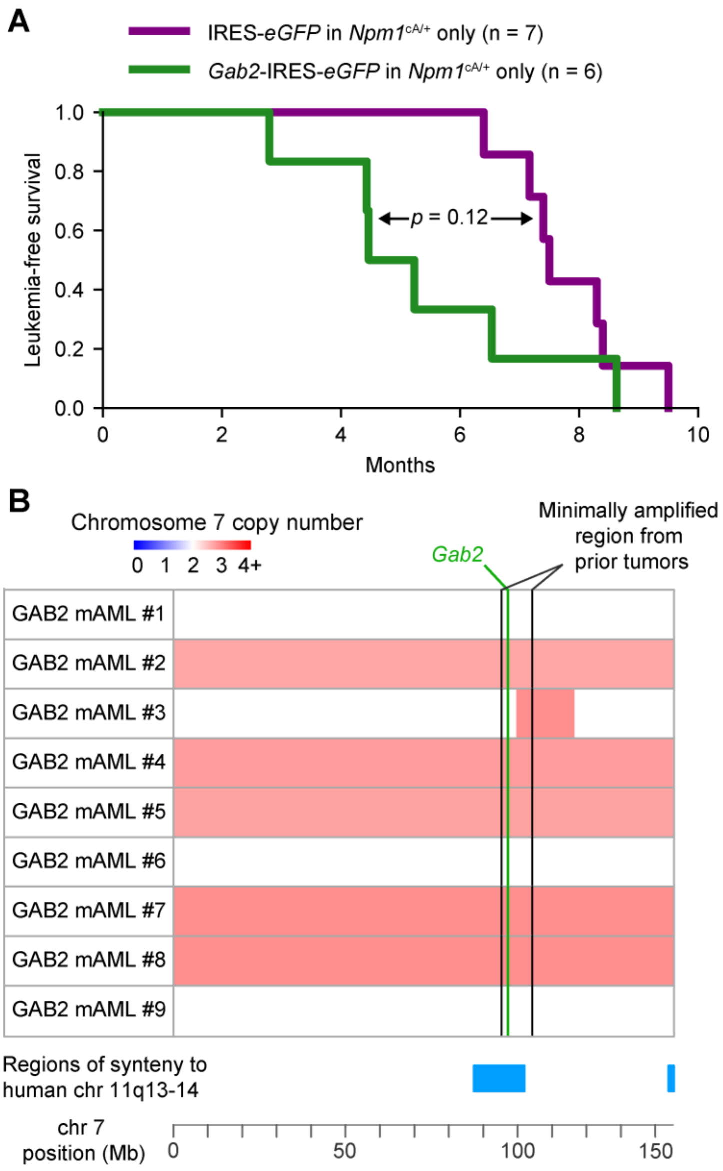

Supplemental Figure 2. Effects of retroviral overexpression of *Gab2* on the development of AML. (A) Mice with the indicated genotypes were monitored for the development of AML. Retroviral overexpression of *Gab2* in the *Npm1*$^{cA/+}$ background shows a non-significant trend towards acceleration of AML development. *p*-value by pairwise log-rank test. **(B)** Copy number variants detected in whole genome sequencing from GAB2 mAMLs (developed in mice transplanted with *Dnmt3a*$^{R878H/+}$ x *Npm1*$^{cA/+}$ bone marrow with *Gab2*-IRES-*eGFP* retroviral overexpression). Note GAB2 mAML #3 has a partial amplification of chr7 that does not include the region of chr7 containing *Gab2* (indicated with green line). The previously identified minimally amplified region as identified in Figure 1C is outlined in black. Regions of synteny with human chromosome 11q13-14 are shown in blue.

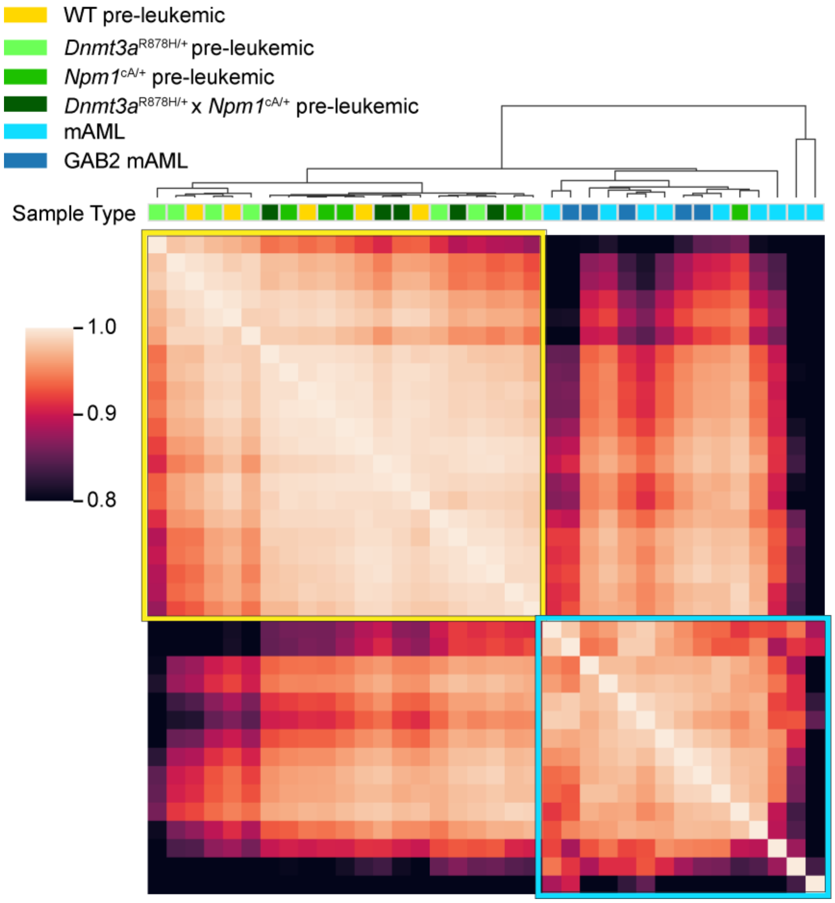

Supplemental Figure 3. Hierarchical clustering of RNA-seq data shows separation of AML and pre-leukemic samples, and intermixing of mAML and GAB2 mAML samples. Hierarchical clustering of bulk RNA sequencing data from pre-leukemic and leukemic samples of the indicated genotypes using the UPGMA method (4) and Pearson correlation of $\log_2$ transformed mRNA abundance profiles as the distance metric. A yellow box highlights the group of primarily pre-leukemic samples, and a blue box highlights the AML samples. Note that mAML and GAB2 mAMLs are intermixed, suggesting their transcriptional similarity.

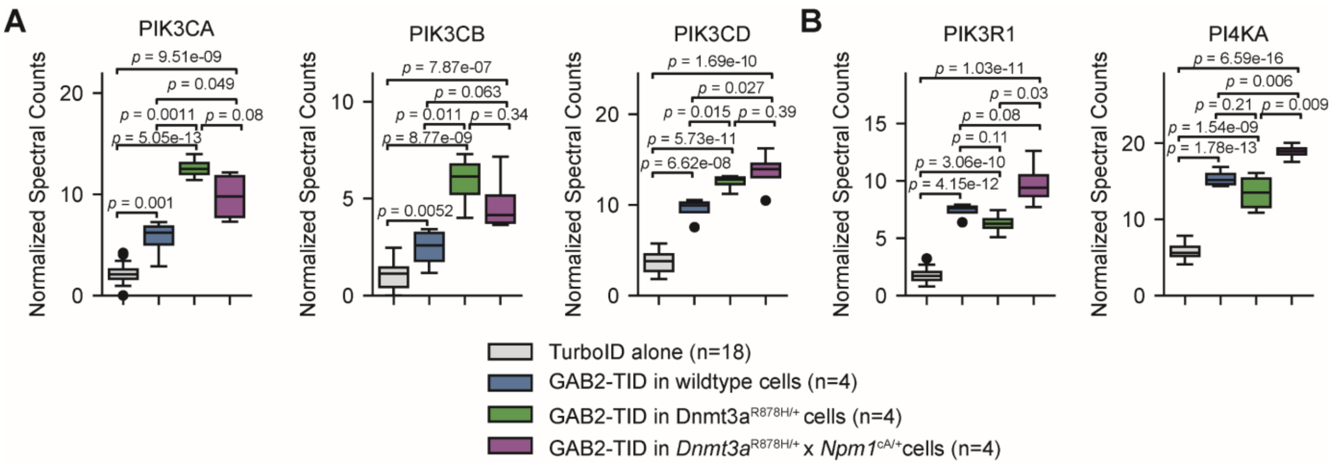

Supplemental Figure 4. The GAB2 protein interactome is shaped by *Dnmt3a*^{R878H} and *Npm1*^{cA} mutations. (A, B) TurboID (TID)-based proximity labeling was performed in primary, lineage-depleted murine bone marrow cells of the indicated genotypes. Normalized spectral counts of the indicated PI3-kinase catalytic subunits (Panel A), regulatory subunit (Panel B, left) or PI4-kinase catalytic subunit (Panel B, right). Boxes are colored based on the TurboID “bait” used, and genotype of the primary murine bone marrow cells transduced. Boxes show medians (line) and extend from the 25th to 75th inter-quartile range (IQR), with whiskers showing 1.5X the IQR and outlier points outside the whiskers. *p*-values were calculated by two-sample t-test and corrected for multiple hypothesis testing using the Benjamini-Hochberg method. Note that GAB2 interactions are genotype-dependent in each case.

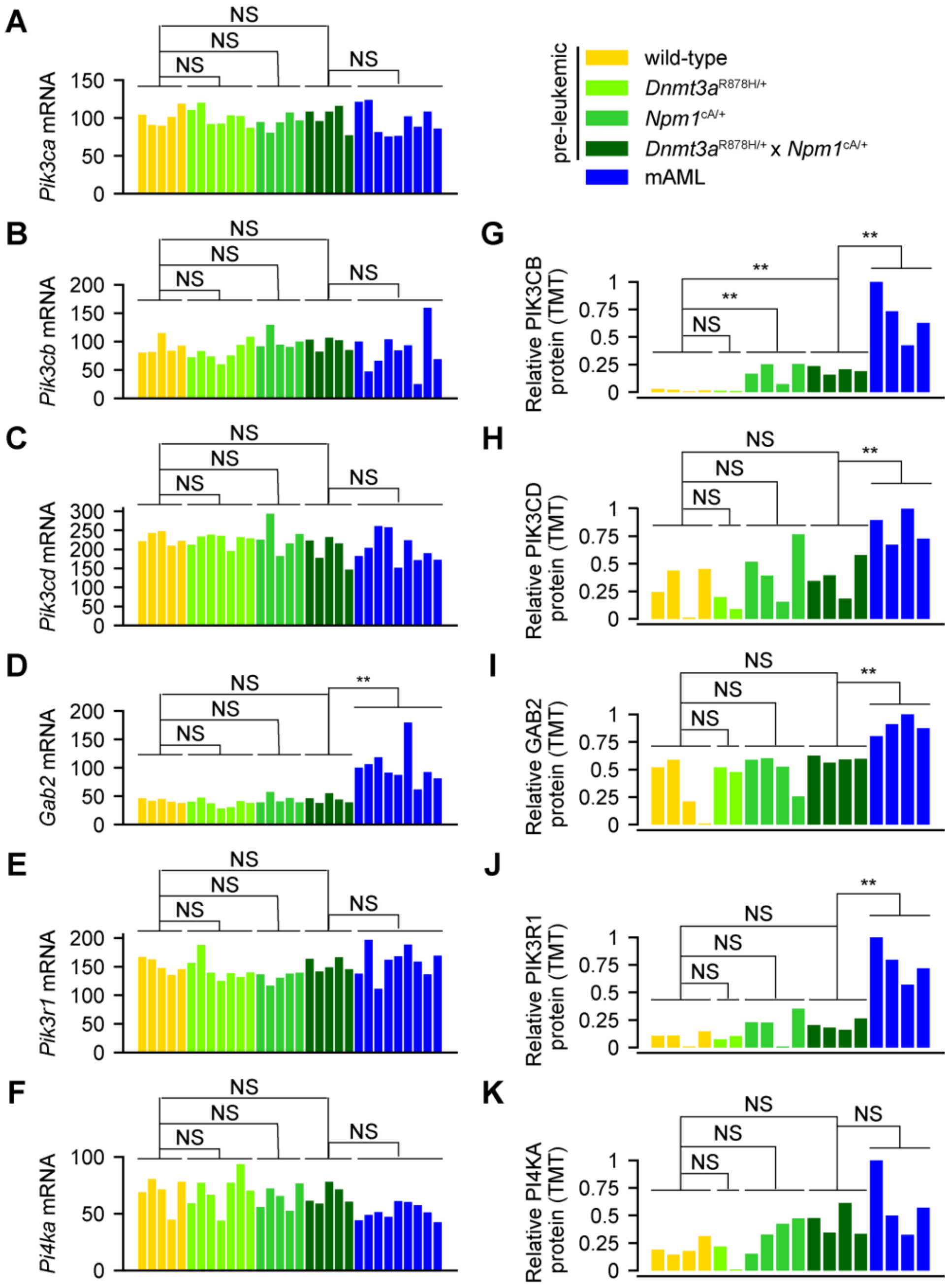

Supplemental Figure 5. mRNA and protein expression of genes with genotype-dependent protein interactions identified by TurboID. (A-F) mRNA expression and **(G-K)** protein abundance from whole bone marrow of pre-leukemic and leukemic (mAML) mice from this model. Each bar represents a measurement from one mouse. For pre-leukemic samples, ** indicates adjusted *p*-value < 0.05 for difference from wild-type by t-test with Benjamini-Hochberg multiple hypothesis correction (note that no significant differences in mRNA expression were found between pre-leukemic bone marrow of any genotype tested and wild-type for any of the displayed genes, each of which showed genotype-dependent TurboID interactions with either NPM1^{cA} or GAB2 as shown in Figure 4 and Supplemental Figure 4). For mAML samples, ** indicates adjusted *p*-value < 0.05 for difference from *Dnmt3a*$^{R878H/+}$ x *Npm1*$^{cA/+}$ by t-test with Benjamini-Hochberg multiple hypothesis correction. For tandem-mass-tag (TMT) proteomics, mAML samples are two biological replicates each of mAML #1 and mAML #3 transplanted into secondary recipients. Relative protein expression values are normalized to lie between 0 and 1 for display (statistical testing performed prior to normalization). Only PIK3CB showed a significant difference between any pre-leukemic genotype and wild-type for the displayed proteins. PIK3CA protein was not detected in these samples (due to limit of detection of this method), and is therefore not shown. Note that PIK3CB, PIK3CD and PIK3R1 do show statistically significant increases in protein levels in AML cells compared to doubly-mutant pre-leukemic bone marrow samples, despite no significant changes in mRNA expression.

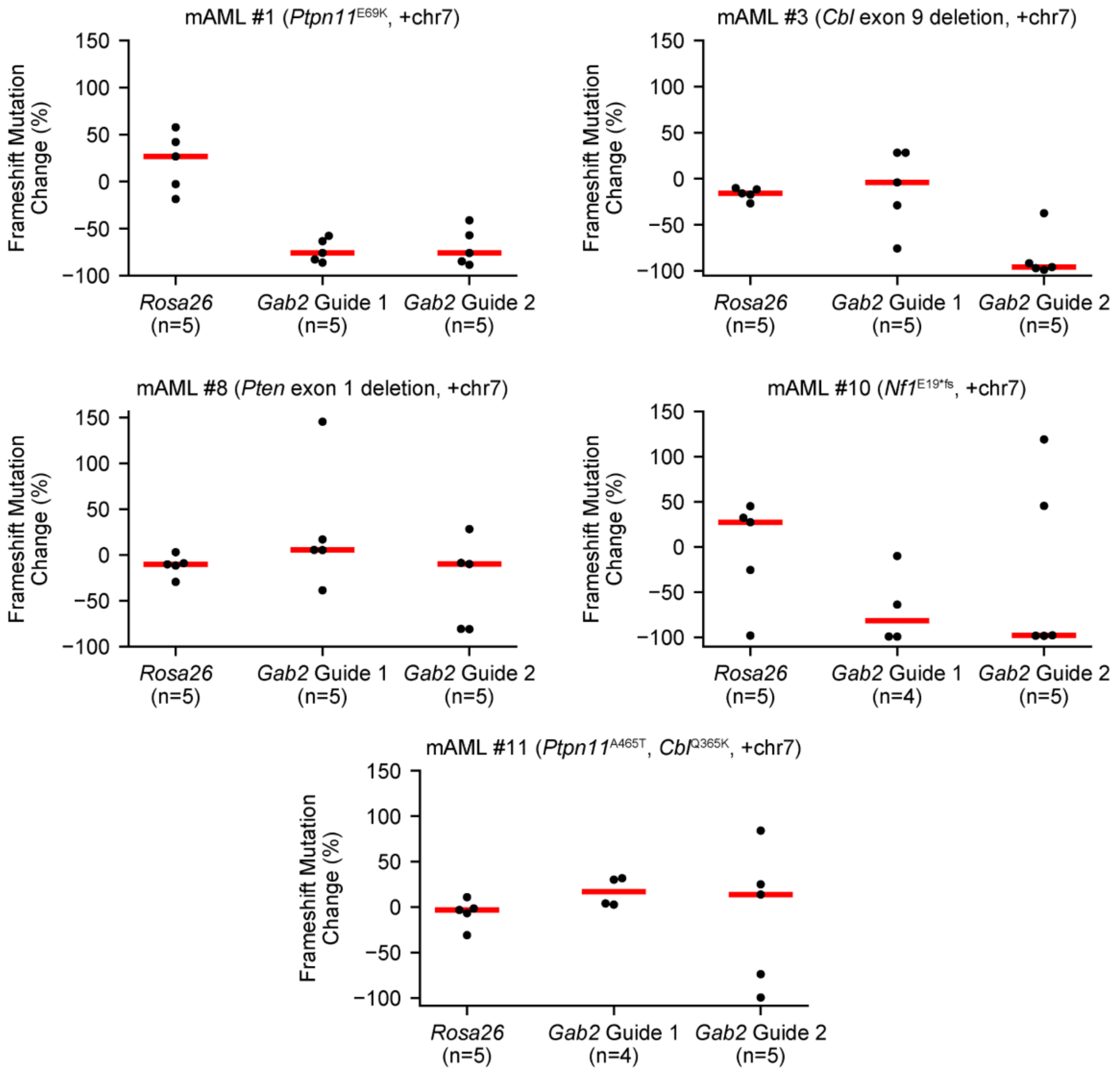

Supplemental Figure 6. Inactivation of *Gab2* mitigates the growth of some fully transformed murine AML cells. *Dnmt3a*$^{R878H/+}$ x *Npm1*$^{cA/+}$ murine AML (mAML) cells were harvested from mouse bone marrow and transfected with Cas9 protein and guide RNAs targeting *Gab2* (two independent guides) or *Rosa26* (negative control). Cells were then transplanted into sublethally irradiated (6Gy) CD45.1+ recipient mice. An aliquot of cells was maintained in vitro for 24-48 hours prior to harvest to measure baseline editing efficiency. Bone marrow from transplanted mice was harvested when mice were moribund. All mice showed >95% CD45.2+ AML cells in the bone marrow at time of harvest. DNA from bone marrow was purified, and targeted PCR-based sequencing of edited loci was performed. Each point represents an individual mouse and shows the percentage change in frameshift mutations detected at time of harvest compared to baseline. Red line indicates the median of each group. These data are the same as presented in Figure 7C, but separated by mAML tumor and guide. The mutations previously identified in each tumor are shown.

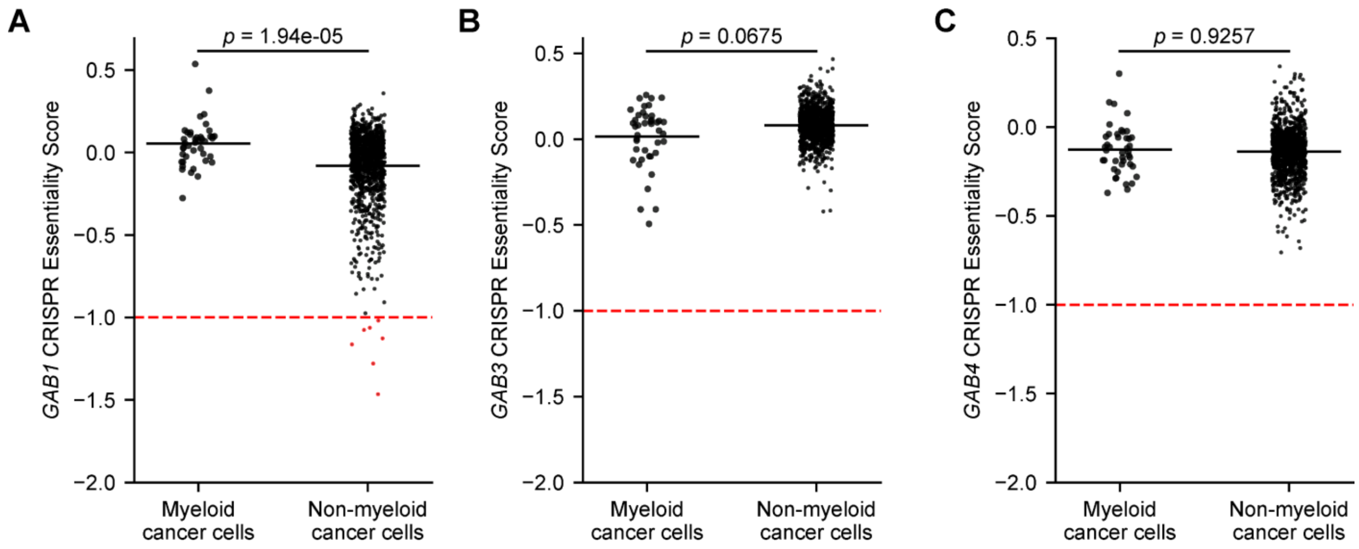

Supplemental Figure 7. Of the GAB-family genes, only *GAB2* is relevant for the survival of human myeloid cancer cell lines. (A-C) CRISPR Essentiality score from the Broad Cancer DepMap CHRONOS dataset in human cancer cell lines for *GAB1* (Panel A), *GAB3* (Panel B), and *GAB4* (Panel C). 0 indicates no effect. Black line indicates the mean of each group. Dotted red line (-1) corresponds to the median value of all pan-essential genes. *p*-value by Wilcoxon rank-sum test.

Captions for Supplemental Tables

Supplemental Table 1: Genomic variants and copy number alterations as detected by whole genome sequencing of murine AMLs arising in *Dnmt3a*$^{R878H/+}$ x *Npm1*$^{cA/+}$ mice (mAML).

Supplemental Table 2: Gene ontology term enrichment for differentially expressed genes (DEGs) in RNA sequencing data, calculated from expression values from mAML samples vs. pre-leukemic *Dnmt3a*$^{R878H/+}$ x *Npm1*$^{cA/+}$ murine bone marrow samples.

Supplemental Table 3: Protein coding genes within the minimally amplified region on murine chromosome 7 (chr7:95301167-104284544).

Supplemental Table 4: Genomic variants and copy number alterations detected by whole genome sequencing of murine AML arising in *Dnmt3a*$^{R878H/+}$ x *Npm1*$^{cA/+}$ mice with retroviral overexpression of *Gab2* (GAB2 mAML).

Supplemental Table 5: Protein coding genes within the minimally amplified region on murine chromosome 7 (chr7: 99644109-104284544).

Supplemental Table 6: Results of differential gene expression analysis using RNA-seq data from GAB2 mAML vs. mAML samples. Differentially expressed genes (FDR < 0.01) are in red.

Supplemental Table 7: 20 proteins with 1) increased protein abundance in mAML tumors, compared to pre-leukemic *Dnmt3a*$^{R878H/+}$ x *Npm1*$^{cA/+}$ bone marrow; 2) no significant change in mRNA expression in mAML tumors, compared to pre-leukemic *Dnmt3a*$^{R878H/+}$ x *Npm1*$^{cA/+}$ bone marrow; 3) significant proximity interaction between NPM1^{cA} and the protein of interest, as determined by TurboID; 4) no significant proximity interaction between wild-type NPM1 and the protein of interest, as measured by TurboID.

Supplemental Table 8: List of 20 genes with an identified PML::RARA binding site within 1 kB of their 5' ends in both mouse and human hematopoietic cells, and increased expression in *PML::RARA* expressing promyelocytes (5).

Supplemental Methods

Generation of *Dnmt3a*$^{R878H/+}$ x *Npm1*$^{cA/+}$ murine AML. Mice with a germline *Dnmt3a*$^{R878H/+}$ allele, generated as previously described (6, 7) were crossed with homozygous *R26*^{FlipoER} mice (Jackson Labs #019016). *Dnmt3a*$^{R878H/+}$ x *R26*$^{FlipoER/+}$ mice were then crossed with *Npm1*$^{frt\text{-}cA}$ knock-in mice (Jackson Labs #033164) to generate *Dnmt3a*$^{R878H/+}$ x *R26*$^{FlipoER/+}$ x *Npm1*$^{frt\text{-}cA/+}$ progeny. Bone marrow from these mice was then transplanted into irradiated (6Gy) CD45.1+ recipient mice (Jackson Labs #002014). After engraftment (4 weeks), recipients were treated with intraperitoneal tamoxifen (75 mg/kg per day for 5 days) to activate the *Npm1*^{cA} allele. Mice were monitored with monthly blood counts and harvested when moribund. AMLs harvested from living mice were transplanted into sublethally irradiated (6Gy), congenic recipients to confirm lethality with secondary transplantation.

Whole genome sequencing and analysis. Whole genome sequencing libraries were created using the Illumina TruSeq PCR-free kit and sequenced with 2x151 bp reads using an Illumina NovaSeq instrument to a mean depth of 31.4X coverage. Somatic variants were called using a CWL pipeline comprehensively described in https://github.com/genome/analysis-workflows/commit/061d3a2fbcd8a1c39c0b38c549e528deb24a9d54. Additional filtering was done to remove artifacts at homopolymer tracts, as well as SNPs present in the original mouse strain. All mutations occurring in cancer-related genes were manually reviewed. Structural variants were called using Manta version 1.6.0 and STAR-fusion 1.11 was used to detect gene fusion events. Both were followed by filtering and manual review. While these callers detected several structural events that were included in Supplemental Table 1, no gene fusions were observed.

Copy number analysis. Copy number calling from targeted sequencing data (8, 9) was performed using the CNVkit package (10) with default parameters, comparing those studies' malignancies to their pooled normals. Copy number data from arrayCGH (11) was analyzed with the DNAcopy package (12) using parameters 'alpha=0.01, min.width=3, undo.splits="sdundo", undo.SD=2'. Copy number from WGS was called using CNVkit. To address hypersegmentation and reference/alignment artifacts, segments with less than 20 probes of support were removed from array/targeted sequencing calls. For WGS data, segments with less than 80 windows of support were removed (predominantly genomic repeats), as were segments with $\log_2$ copy number greater than 0.8 and size less than 500 windows (predominantly heterochromatin). In all data, adjacent segments with copy

number difference of less than 0.1 were merged. Segments intersecting at least 50% with gaps in the mouse reference genome were removed, as were selected artifacts after manual review.

RNA sequencing and analysis. Total RNA-seq was performed using the Illumina TruSeq Stranded Total RNA Library Kit on deoxyribonuclease-treated RNA and sequenced on the NovaSeq6000 platform, with 2x151 bp reads. Differentially expressed genes were called using the edgeR package, version 3.20.9 (13).

Retroviral transductions. As described previously (14), retroviruses were prepared by transfection of GP2-293 cells (Takara) with target plasmids, and the VSVg packaging plasmid using TransIT-LT1 (Mirus Bio) or Lipofectamine 3000 (Invitrogen). Retroviruses were adsorbed to tissue culture plates coated with RetroNectin (Takara), and cells were spun onto the adsorbed retroviral particles for transfection.

Cell culture of lineage negative primary murine bone marrow. Cells were cultured in RPMI 1640 (Gibco) containing 15% FBS (R&D Systems), penicillin-streptomycin (Gibco), 100 ng/mL SCF, 10 ng/mL thrombopoietin, 50 ng/mL FLT3L, and 6 ng/mL IL-3 (PeproTech). Lineage depletion was performed using the Direct Lineage Cell Depletion Kit and the autoMACS magnetic cell separator (Miltenyi Biotec).

Western blotting. Western blots were performed using the ProteinSimple Jess system (Bio-techne) using the following antibodies: GAB2 (Abcam ab235932), PAK1 (Abcam ab223849), β-actin (Cell Signaling #4970), AKT (Cell Signaling #2920), phospho-AKT Ser473 (Cell Signaling #4060), ERK1/2 (Cell Signaling #4695) and phospho-ERK1/2 Thr202/Tyr204 (Cell Signaling #4370). For phospho-blotting, cells were cultured for 24 hours in serum-reduced media (RPMI 1640 with 0.2% FBS and penicillin/streptomycin, no cytokines) prior to harvest. Cells were counted and lysed at 10^6 cells per 100 uL in NuPAGE LDS buffer (Invitrogen) with protease/phosphatase inhibitors (Cell Signaling #5872), prior to loading on the Jess apparatus.

Multiparameter flow cytometry. Cells were stained with antibodies to CD16/32, B220, CD11b, CD3, CD19, c-KIT, CD11c, CD45.2, CD71, NK1.1, CD150, TER119, CD45.1, FLT3, SCA-1, CD48, CD116, CD34, IL-7RA, GR1 and CD4, and with Zombie UV (Biolegend) viability stain as described previously (7). Flow cytometry for the named markers and GFP was performed using the Cytek Aurora Spectral Cytometer, and data were analyzed using OMIQ software. Cells were characterized using canonical surface markers: CD3 (T cells), CD11b (myeloid cells), CD19 (B cells), CD71 and TER119 (erythroid cells). Lineage negative cells were negative for CD3, CD19, GR-

1, CD11b, TER119, and NK1.1. UMAP visualization procedure performed using all markers, excluding GFP and viability stain.

TurboID proximity labeling. TurboID proximity labeling and mass spectrometry were performed exactly as described (14).

Mass spectrometry. Tandem-mass-tag (TMT) and label-free-quantification (LFQ) proteomics were performed on unfractionated bone marrow samples from individual mice, as previously described (15).

CRISPR of primary murine AML cells. Bone marrow was harvested from mice with engrafted secondary or tertiary AMLs. Flow cytometry confirmed that >90% of bone marrow cells were of AML origin at harvest. Guide crRNA (IDT) was annealed to tracrRNA (IDT) per manufacturer's instructions, and incubated with Cas9 protein to form the RNP complex. Cells were electroporated with the RNP complex using a Lonza Nucleofector X, or a Bulldog Bio Nepa21. Cells were then split into two aliquots, with >100K cells maintained in vitro for 24-48 hours to measure baseline editing efficiency, and the remainder split equally for transplantation into sublethally irradiated (6 Gy) CD45.1 recipient mice (0.5 – 1 million cells per mouse). Guides from IDT were purchased for *Rosa26* (TGCAAGTTGAGTCCATCCGC), and *Gab2* (Guide 1 = ACGACGTGGTGTGTACCGGC and Guide 2 = GGACACTGTATCAACGGCGT).

Culture of primary human AML samples. As described previously (16, 17), cryopreserved samples were thawed and plated on irradiated HS-27 stromal cells in media containing DMEM, 20% FBS, 1:1000 beta-mercaptoethanol, penicillin-streptomycin (Gibco), 100 ng/mL SCF, 10 ng/mL FLT3L, 10 ng/mL TPO, 10 ng/mL IL-3 and 20 ng/mL IL-6 (PeproTech). Media was changed 3X weekly.

Human CD34+ cell isolation from cord blood. Deidentified human cord blood samples were obtained at Washington University; mothers were consented prior to scheduled C-sections as per WashU IRB #202104011, and samples were obtained without interfering with standard delivery practices. Mononuclear cells were isolated using Ficoll and Sepmate-50 conicals per manufacturer's instructions. CD34+ cells were isolated using the CD34 microbead kit and autoMACS magnetic cell separator (Miltenyi Biotec). Cells were maintained in vitro in media containing DMEM, 20% FBS, 1:1000 beta-mercaptoethanol, penicillin-streptomycin (Gibco), 100 ng/mL SCF, 10 ng/mL FLT3L, 10 ng/mL TPO, 10 ng/mL IL-3 and 20 ng/mL IL-6 (PeproTech).

CRISPR of K562 cells, human CD34+ cord blood cells, primary human AML cells and xenografting. K562 cells were obtained from ATCC. Primary human AML cells were thawed from stably preserved cryovials of leukapheresis products, or peripheral blood samples from human AML patients, collected with informed consent using an IRB approved protocol (WashU #201011766). Cells were transfected using the Bulldog Bio Nepa21 with *Cas9* mRNA and sgRNA purchased from IDT to *AAVS1* (GCCACUAGGGACAGGAUGU) or *GAB2* (Guide 1 = AUACUCCUGAGGUGCGCG, Guide 2 = GUAAAAGGUGCGUUCACG). For xenografting experiments, an aliquot of cells was maintained in vitro for 24-48 hours to measure baseline editing efficiency. The remaining cells were split equally (0.5 – 1 million cells per mouse) and transplanted into sublethally irradiated (2Gy) NSG-SGM3 mice (Jackson Labs #013062). Mice were monitored monthly for engraftment of human AML cells using flow cytometry of blood samples. Once engrafted (>1% of peripheral blood human CD45+, human CD33+; all AMLs used were CD33+ at baseline), bone marrow was harvested and flow sorted for human AML cells prior to extraction of DNA for sequencing.

Methylcellulose-based culture of murine HSPCs and APL. Cells were cultured in Methocult GF M3434 media (StemCell Technologies), supplemented with 50 ng/mL FLT3L, as described previously (18, 19).

Supplemental References

1. Nishida K, Yoshida Y, Itoh M, Fukada T, Ohtani T, Shirogane T, et al. Gab-family adapter proteins act downstream of cytokine and growth factor receptors and T- and B-cell antigen receptors. *Blood.* 1999;93(6):1809-16.
2. Craddock BL, and Welham MJ. Interleukin-3 induces association of the protein-tyrosine phosphatase SHP2 and phosphatidylinositol 3-kinase with a 100-kDa tyrosine-phosphorylated protein in hemopoietic cells. *J Biol Chem.* 1997;272(46):29281-9.
3. Gu H, Pratt JC, Burakoff SJ, and Neel BG. Cloning of p97/Gab2, the major SHP2-binding protein in hematopoietic cells, reveals a novel pathway for cytokine-induced gene activation. *Mol Cell.* 1998;2(6):729-40.
4. Sokal RR, and Michener C. University of Kansas. *A statistical method for evaluating systematic relationships.* 1958;28.
5. Katerndahl CDS, Rogers ORS, Day RB, Xu Z, Helton NM, Ramakrishnan SM, et al. PML::RARA and GATA2 proteins interact via DNA templates to induce aberrant self-renewal in mouse and human hematopoietic cells. *Proc Natl Acad Sci U S A.* 2024;121(18):e2317690121.
6. Guryanova OA, Shank K, Spitzer B, Luciani L, Koche RP, Garrett-Bakelman FE, et al. DNMT3A mutations promote anthracycline resistance in acute myeloid leukemia via impaired nucleosome remodeling. *Nat Med.* 2016;22(12):1488-95.
7. Smith AM, LaValle TA, Shinawi M, Ramakrishnan SM, Abel HJ, Hill CA, et al. Functional and epigenetic phenotypes of humans and mice with DNMT3A Overgrowth Syndrome. *Nat Commun.* 2021;12(1):4549.
8. Loberg MA, Bell RK, Goodwin LO, Eudy E, Miles LA, SanMiguel JM, et al. Sequentially inducible mouse models reveal that Npm1 mutation causes malignant transformation of Dnmt3a-mutant clonal hematopoiesis. *Leukemia.* 2019;33(7):1635-49.
9. SanMiguel JM, Eudy E, Loberg MA, Miles LA, Stearns T, Mistry JJ, et al. Cell origin-dependent cooperativity of mutant Dnmt3a and Npm1 in clonal hematopoiesis and myeloid malignancy. *Blood Adv.* 2022;6(12):3666-77.
10. Talevich E, Shain AH, Botton T, and Bastian BC. CNVkit: Genome-Wide Copy Number Detection and Visualization from Targeted DNA Sequencing. *PLoS Comput Biol.* 2016;12(4):e1004873.
11. Dovey OM, Cooper JL, Mupo A, Grove CS, Lynn C, Conte N, et al. Molecular synergy underlies the co-occurrence patterns and phenotype of NPM1-mutant acute myeloid leukemia. *Blood.* 2017;130(17):1911-22.
12. Venkatraman ES, and Olshen AB. A faster circular binary segmentation algorithm for the analysis of array CGH data. *Bioinformatics.* 2007;23(6):657-63.
13. Robinson MD, McCarthy DJ, and Smyth GK. edgeR: a Bioconductor package for differential expression analysis of digital gene expression data. *Bioinformatics.* 2010;26(1):139-40.
14. Day RB, Hickman JA, Xu Z, Katerndahl CD, Ferraro F, Ramakrishnan SM, et al. Proteogenomic analysis reveals cytoplasmic sequestration of RUNX1 by the acute myeloid leukemia-initiating CBFB::MYH11 oncofusion protein. *J Clin Invest.* 2023;134(4).
15. Kramer MH, Zhang Q, Sprung R, Day RB, Erdmann-Gilmore P, Li Y, et al. Proteomic and phosphoproteomic landscapes of acute myeloid leukemia. *Blood.* 2022;140(13):1533-48.
16. Klco JM, Spencer DH, Miller CA, Griffith M, Lamprecht TL, O'Laughlin M, et al. Functional heterogeneity of genetically defined subclones in acute myeloid leukemia. *Cancer Cell.* 2014;25(3):379-92.
17. Klco JM, Spencer DH, Lamprecht TL, Sarkaria SM, Wylie T, Magrini V, et al. Genomic impact of transient low-dose decitabine treatment on primary AML cells. *Blood.* 2013;121(9):1633-43.
18. Katerndahl CDS, Rogers ORS, Day RB, Cai MA, Rooney TP, Helton NM, et al. Tumor suppressor function of Gata2 in acute promyelocytic leukemia. *Blood.* 2021;138(13):1148-61.
19. Cole CB, Verdoni AM, Ketkar S, Leight ER, Russler-Germain DA, Lamprecht TL, et al. PML-RARA requires DNA methyltransferase 3A to initiate acute promyelocytic leukemia. *J Clin Invest.* 2016;126(1):85-98.
