# Supplementary material for: Overexpression of the signaling coordinator GAB2 can play an important role in acute myeloid leukemia progression
Source: J Clin Invest. 2025 Aug 7;135(21):e195929. doi: 10.1172/JCI195929 (PMC12578389; doi:10.1172/JCI195929)
Supplement: Unedited blot and gel images [file jci-135-195929-s151.pdf]

Full unedited blots

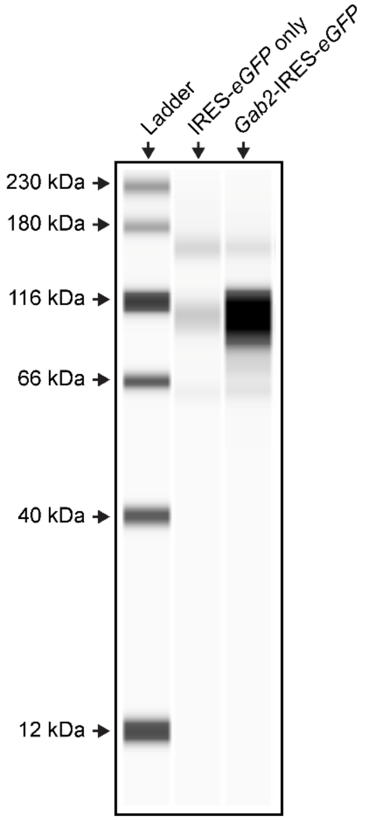

Full unedited blot from ProteinSimple Jess western blotting system for Supplemental Figure 1A, top (anti-GAB2 antibody)

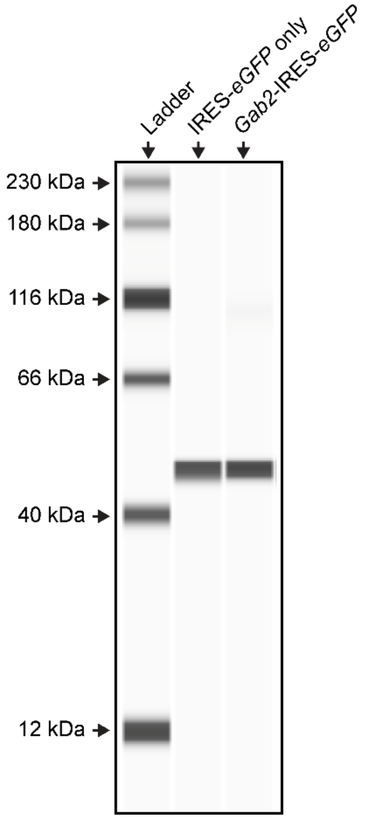

Full unedited blot from ProteinSimple Jess western blotting system for Supplemental Figure 1A, bottom (anti-Actin antibody)

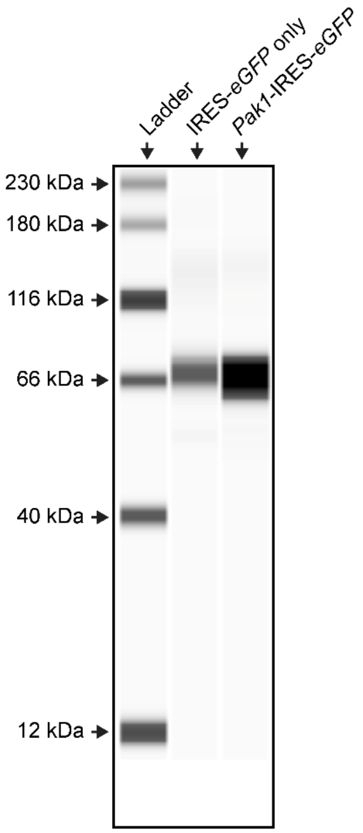

Full unedited blot from ProteinSimple Jess western blotting system for Supplemental Figure 1B, top (anti-PAK1)

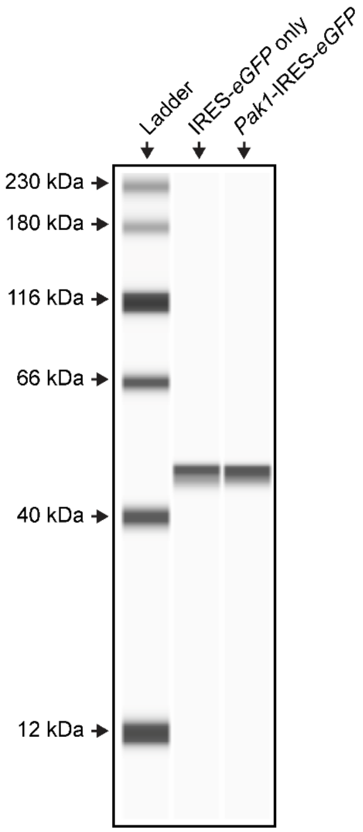

Full unedited blot from ProteinSimple Jess western blotting system for Supplemental Figure 1B, bottom (anti-Actin antibody)

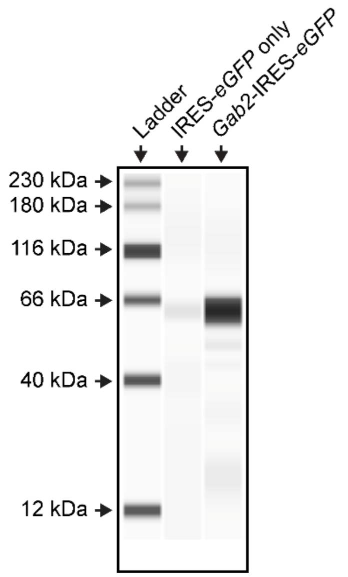

Full unedited blot from ProteinSimple Jess western blotting system for Figure 4A, top (anti-pAKT Ser473 antibody)

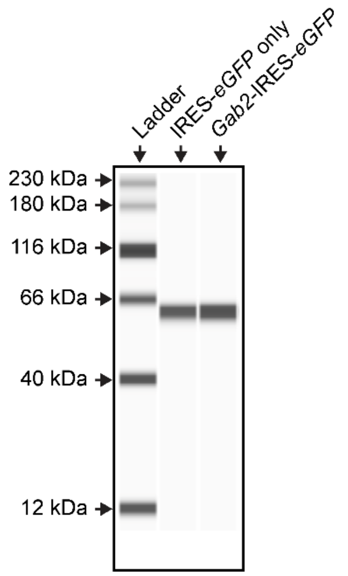

Full unedited blot from ProteinSimple Jess western blotting system for Figure 4A, bottom (anti-AKT antibody)

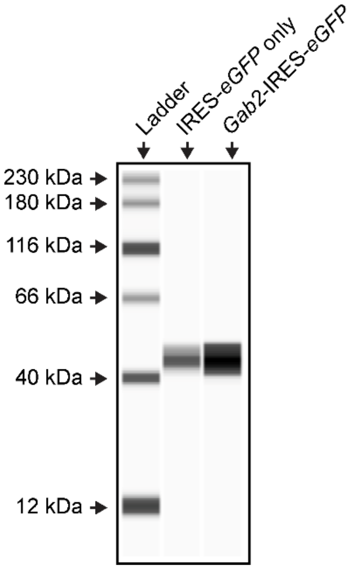

Full unedited blot from ProteinSimple Jess western blotting system for Figure 4B, top (anti pERK1/2 Thr202/Tyr204 antibody)

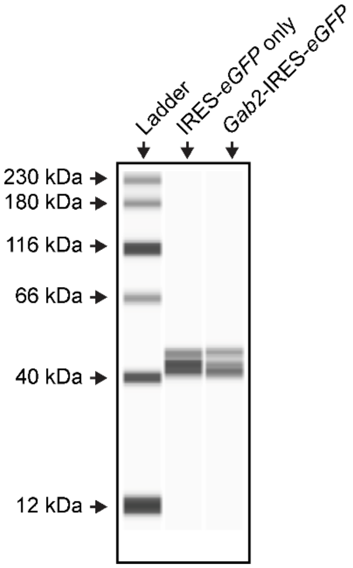

Full unedited blot from ProteinSimple Jess western blotting system for Figure 4B, bottom (anti-ERK1/2 antibody)
